# Discovery of VU6016235: A Highly Selective, Orally Bioavailable, and Structurally Distinct Tricyclic M_4_ Muscarinic Acetylcholine Receptor Positive Allosteric Modulator (PAM)

**DOI:** 10.1021/acschemneuro.4c00465

**Published:** 2024-09-24

**Authors:** Julie L. Engers, Logan A. Baker, Sichen Chang, Vincent B. Luscombe, Alice L. Rodriguez, Colleen M. Niswender, Hyekyung P. Cho, Michael Bubser, Analisa Thompson Gray, Carrie K. Jones, Weimin Peng, Jerri M. Rook, Thomas M. Bridges, Olivier Boutaud, P. Jeffrey Conn, Darren W. Engers, Craig W. Lindsley, Kayla J. Temple

**Affiliations:** † Warren Center for Neuroscience Drug Discovery, Vanderbilt University, Nashville, Tennessee 37232, United States; ‡ Department of Pharmacology, Vanderbilt University School of Medicine, Nashville, Tennessee 37232, United States; § Department of Chemistry, Vanderbilt University, Nashville, Tennessee 37232, United States; ∥ Department of Biochemistry, Vanderbilt University, Nashville, Tennessee 37232, United States; ⊥ Vanderbilt Kennedy Center, Vanderbilt University School of Medicine, Nashville, Tennessee 37232, United States; # Vanderbilt Brain Institute, Vanderbilt University School of Medicine, Nashville, Tennessee 37232, United States

**Keywords:** Muscarinic acetylcholine receptor (mAChR), Muscarinic acetylcholine receptor subtype 4 (M_4_), Positive allosteric modulator (PAM), Structure−activity relationship (SAR), Schizophrenia, Parkinson’s disease, Alzheimer’s disease

## Abstract

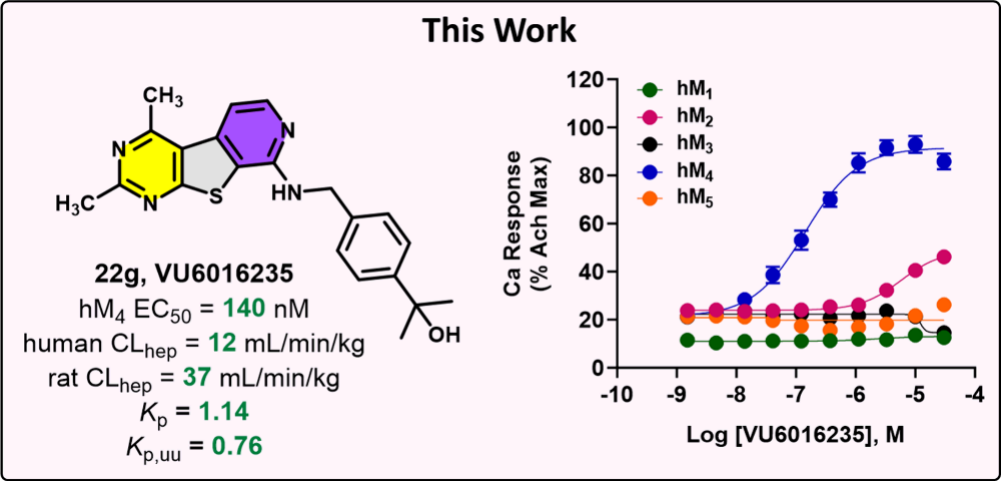

Herein, we report structure–activity relationship (SAR) studies to develop novel tricyclic M_4_ PAM scaffolds with improved pharmacological properties. This endeavor involved a “tie-back” strategy to replace a 5-amino-2,4-dimethylthieno[2,3-*d*]pyrimidine-6-carboxamide core, which led to the discovery of two novel tricyclic cores. While both tricyclic cores displayed low nanomolar potency against both human and rat M_4_ and were highly brain-penetrant, the 2,4-dimethylpyrido[4′,3′:4,5]thieno[2,3-*d*]pyrimidine tricycle core provided lead compound, **VU6016235**, with an overall superior pharmacological and drug metabolism and pharmacokinetics (DMPK) profile, as well as efficacy in a preclinical antipsychotic animal model.

## Introduction

**Figure 1:**
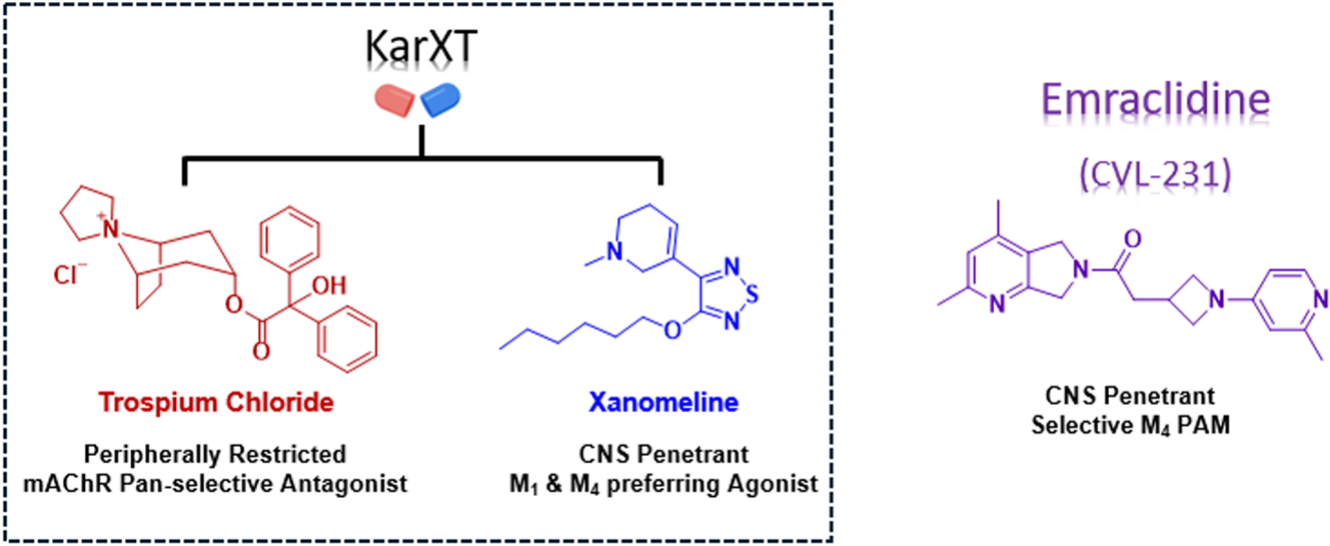
Structures of clinically advanced M_4_-targeting therapeutics.

Muscarinic acetylcholine receptors (mAChRs) are G protein-coupled receptors that have been linked to a variety of central nervous system functions, including cognition, motor control, and sleep–wake architecture.^1^ Muscarinic acetylcholine receptor subtype 4 (M_4_) positive allosteric modulators (PAMs) have gained interest as a novel therapeutic target for the treatment of behavioral and cognitive disturbances associated with both schizophrenia and Alzheimer’s disease (AD), as well as other neurological disorders, such as Parkinson’s disease (PD) and Huntington’s disease (HD).^2−8^ Xanomeline, an M_1_/M_4_-preferring agonist, was evaluated in clinical trials for the treatment of AD and schizophrenia. Unfortunately, adverse cholinergic side effects, associated with a lack of receptor subtype selectivity, halted xanomeline’s clinical development. Nevertheless, data obtained from these trials further validated the muscarinic cholinergic system as a treatment for the psychosis and behavioral disturbances observed in both Alzheimer’s and schizophrenia patients.^9,10^ To overcome these side effects, Karuna Therapeutics (acquired by Bristol Myers Squibb) developed KarXT, which coadministers xanomeline with a pan-selective peripheral mAChR antagonist (trospium chloride) to combat the adverse events associated with xanomeline administration alone (Figure 1).^11^ The New Drug Application (NDA) for KarXT was accepted for review by the FDA in late 2023.^12^ Recently, a selective M_4_ PAM was shown to not only be efficacious in preclinical assays but also exhibited fewer and less severe adverse cholinergic-related side effects when compared to rats treated with xanomeline.^13^ Thus, data suggest that development of a receptor subtype-selective M_4_ PAM could result in improved safety profiles. Emraclidine (CVL-231), a selective M_4_ PAM developed by Cerevel Therapeutics, is currently undergoing clinical trials (Figure 1).^14−20^

**Figure 2:**
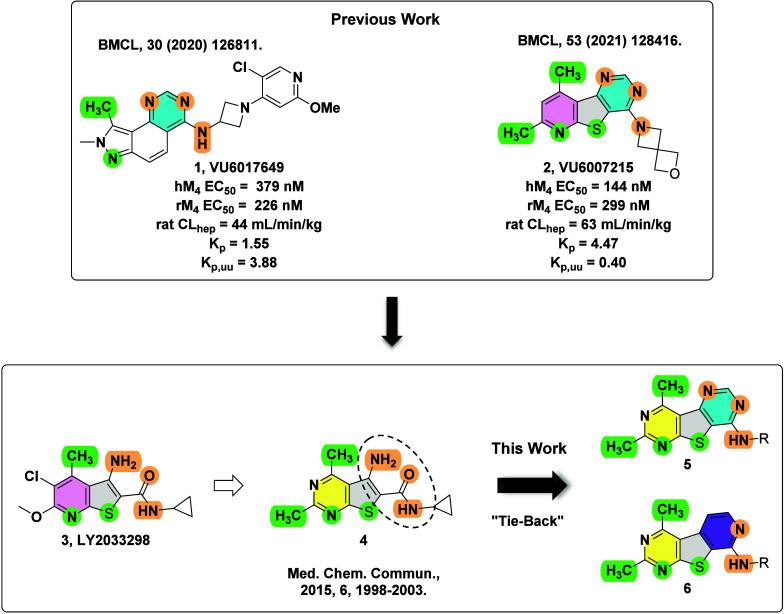
Exploration of novel tricyclic cores as M_4_ PAMs revealed two unique M_4_ PAM tricyclic chemotypes: 7,9-dimethylthieno[2,3-d:4,5-*d*′]dipyrimidine core (**5**) and 2,4-dimethylpyrido[4′,3′:4,5]thieno[2,3-*d*]pyrimidine core (**6**).

Our lab has heavily focused on developing novel, selective M_4_ PAM chemotypes bereft of the traditional β-amino carboxamide moiety historically believed to be essential for M_4_ PAM activity (Figure 2, dashed circle).^21−27^ This key pharmacophore gave rise to M_4_ PAMs with poor solubility, species potency discrepancies, and poor brain exposure.^28−37^ Most recently, our laboratory disclosed a novel 5,6,6-tricyclic scaffold and a novel 6,5,6-tricyclic scaffold that still afforded potent and CNS-penetrant M_4_ PAMs (Figure 2, **VU6007215** and **VU6017649**).^24,25^ To identify additional novel M_4_ PAM chemotypes, we elected to further explore these tricyclic scaffolds. An historic β-amino carboxamide-containing M_4_ PAM (**4**), developed by the Capuano laboratory, formed the base of our novel tricycles.^38^ This exercise resulted in the discovery of a novel M_4_ PAM chemotype containing a 7,9-dimethylthieno[2,3-*d*:4,5-*d*′]dipyrimidine core, **5**. Further exploration revealed a second, novel, tricyclic M_4_ PAM chemotype containing a 2,4-dimethylpyrido[4′,3′:4,5]thieno[2,3-*d*]pyrimidine core, **6**. This body of work details the development of these two novel M_4_ PAM chemotypes.

## Results and Discussion

**Scheme 1:**
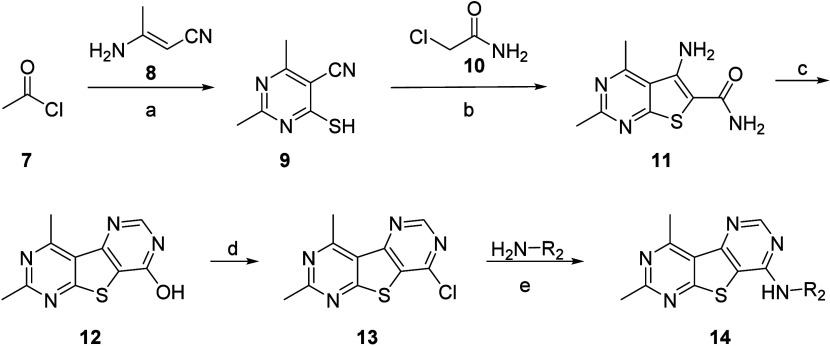
Synthesis of M_4_ PAM Analogues **14** Reagents and conditions: (a) **8**, NH_4_SCN, 1,4-dioxane, 110 °C, 2 h, 46%; (b) **10**, K_2_CO_3_, *N*-methyl-2-pyrrolidone (NMP), microwave-irradiated at 60 °C for 30 min, then microwave-irradiated at 100 °C for 1 h, 43%; (c) CH(OEt)_3_, 150 °C, 3 h, 99%; (d) POCl_3_, TEA, 1,2-dichloroethane (DCE), 110 °C, 3 h, 81%; (e) amine, *N*,*N*-diisopropylethylamine (DIEA), NMP, 60 °C for 2 h or microwave-irradiated at 100 °C for 10 min, 30–77%.

The synthesis of tricyclic core **5** began with reacting commercially available acyl chlorides **7**, 3-aminocrotonitrile (**8**), and ammonium thiocyanate to afford the tetra-substituted pyrimidine **9** (Scheme 1). Similar to published protocols, treatment with 2-chloroacetamide (**10**) and potassium carbonate followed by irradiation in a microwave reactor generated carboxamide **11**.^39^ Treatment of **11** with trimethylorthoformate under heat afforded pyrimidone intermediate **12**. Pyrimidone **12** was then converted into chloride **13** with POCl_3_, which then readily underwent nucleophilic aromatic substitution with a variety of amines to yield desired analogues **14**. For this exercise, we decided to forego exploring amino azetidines of past M_4_ PAMs because of their potential metabolic instability.^40^ Instead, we focused on examining small aliphatic amines, which typically provided potent M_4_ PAMs, as well as substituted benzylamines, which could aid in increasing solubility.

Select analogues **14** were screened against human M_4_ (hM_4_) to determine EC_50_ values with results highlighted in Table 1. These results emphasize the importance of the amine tail on potency. In the context of the 7,9-dimethylthieno[2,3-*d*:4,5-*d’*]dipyrimidine core (**5**), the benzyl amine-containing analogues **14j** and **14k** both have hM_4_ EC_50_ values > 1 μM. However, several of the small aliphatic amine tails provided compounds with hM_4_ EC_50_ values < 500 nM (**14g**, hM_4_ EC_50_ = 290 nM; **14h**, hM_4_ EC_50_ = 100 nM; and **14i**, hM_4_ EC_50_ = 160 nM). It was noticed that minor modifications, such as fluorine substitutions on the pyrrolidine (**14e** vs **14g**), led to a 2.2-fold loss in potency. Moreover, removal of the oxygen from the morpholine analogue **14b** (hM_4_ EC_50_ = 5.6 μM) to give the piperidine analogue **14a** (hM_4_ EC_50_ > 30 μM) resulted in a loss of activity. Additionally, substituting the 2-oxa-6-azaspiro[3.3]heptane tail of **14c** (hM_4_ EC_50_ = 1.6 μM) with the 6,6-difluoro-2-azaspiro[3.3]heptane tail of **14h** (hM_4_ EC_50_ = 100 nM) resulted in a 16-fold increase in functional human M_4_ potency. Interestingly, we also observed an 11-fold loss of potency when comparing the pyridine predecessor compound **VU6007215** (Figure 1; hM_4_ EC_50_ = 144 nM) to the pyrimidine compound **14c**. One could speculate that this result is due to the difference in the tricyclic cores; however, other amine tails were nearly equipotent [e.g., pyrrolidine (**14g**)].^25^ These results reiterate the importance the amine tail can have on potency, particularly in combination with different tricycles.

**Table 1:**
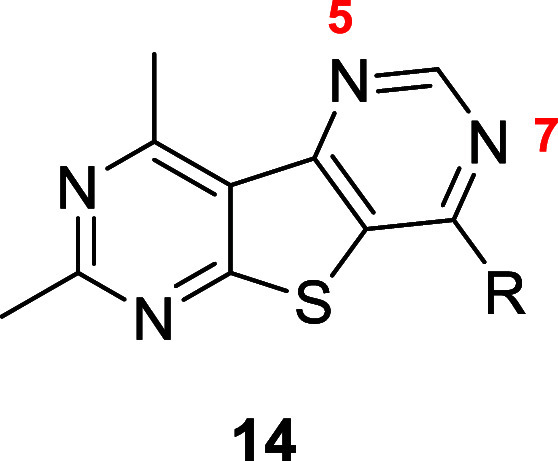

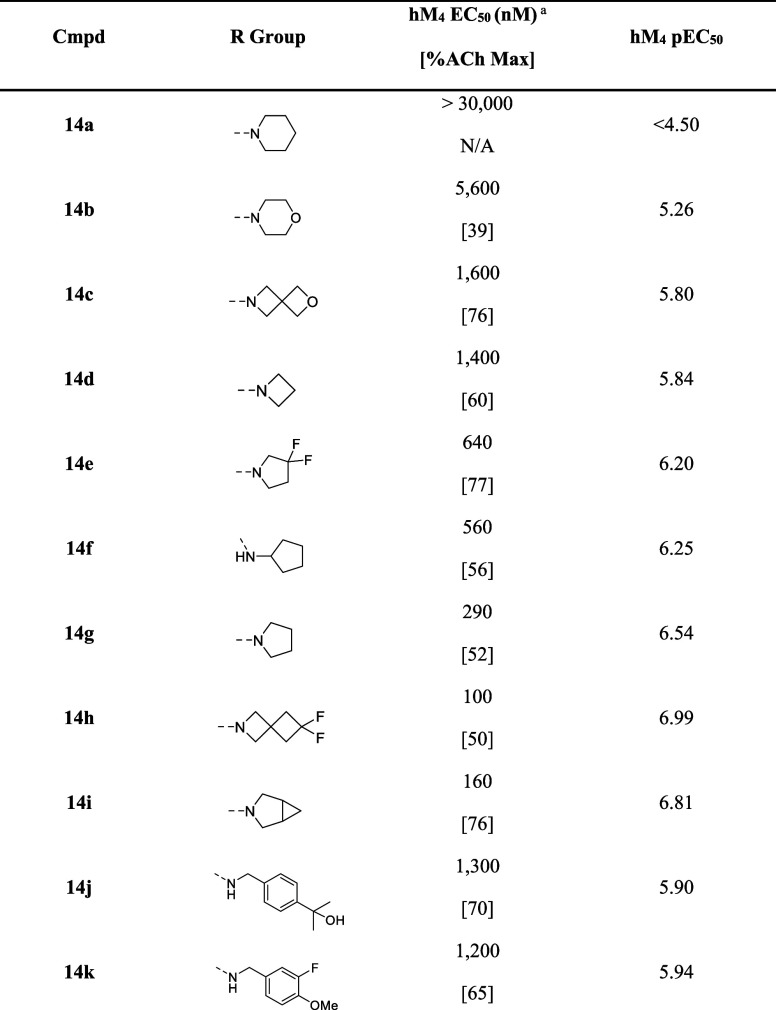
Structures and Activities for Analogues **14**

a Calcium mobilization assays with hM_4/Gqi5_-CHO cells were performed in the presence of an EC_20_ fixed concentration of acetylcholine. EC_50_ values for hM_4_ represent at least one experiment performed in triplicate.

**Scheme 2:**
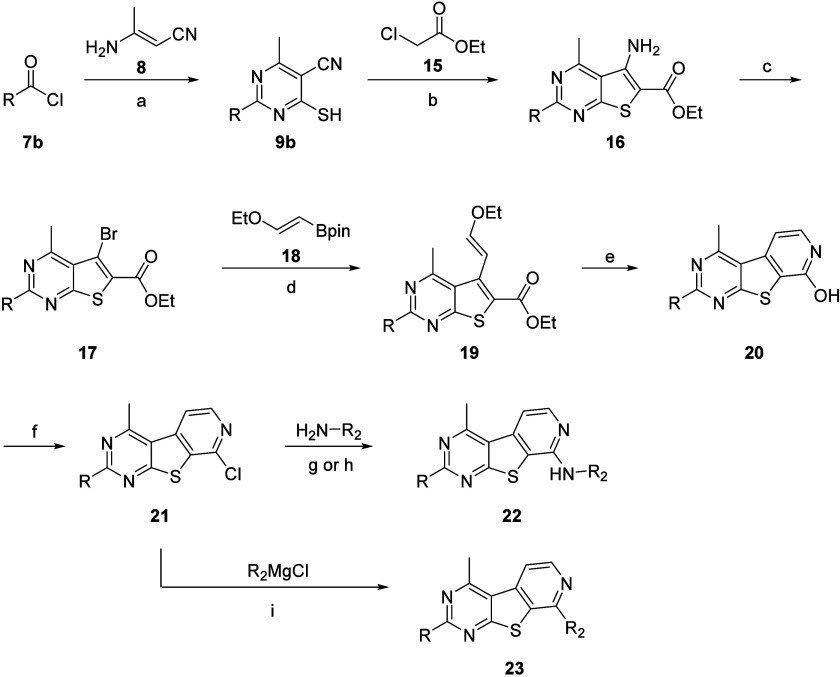
Synthesis of M_4_ PAM Analogues **22** and **23** Reagents and conditions: (a) **8**, NH_4_SCN, 1,4-dioxane, 110 °C, 2 h, 21–42%; (b) **15**, Na_2_CO_3_, DMF, 45 °C, 3 h, 52–91%; (c) CuBr_2_, ^*t*^BuONO, acetonitrile (ACN), 1 h, 40–75%; (d) **18**, Cs_2_CO_3_, Pd(dppf)Cl_2_, 1,4-dioxanes/H_2_O (10:1), 80 °C, 18 h, 44–76%; (e) (i) TFA, 120 °C, 2 h; (ii) NH_4_OH, 100 °C, 4.5 h, 30–88% over two steps; (f) POCl_3_, microwave-irradiated at 120 °C, 30 min, 13–92%; (g) aliphatic amine, DIEA or K_2_CO_3_, NMP, microwave-irradiated at 120–180 °C, 0.5–2 h, 8–20%; (h) benzyl amine, Pd_2_(dba)_3_, Xantphos, Cs_2_CO_3_, 1,4-dioxane, 110 °C, 6–18 h, 14–51%; (i) R_2_MgCl, Fe(acac)_3_, THF/NMP (5:1), 1–18 h, 31–41%.

We next turned our attention to evaluating the relevance of the pyrimidine nitrogen at the 5-position by synthesizing analogues **22** and **23**. Previously, conversion of this pyrimidine ring into a pyridine ring was not detrimental to hM_4_ activity; rather, several analogues displayed increased potency.^41^ To determine if this trend proved true for our current scaffold, we synthesized analogues with the tricyclic core **6**. The synthesis was accomplished by first treating intermediate **9b** with ethyl 2-chloroacetate (**15**) and sodium carbonate to generate carboxylate **16** (Scheme 2). A copper-catalyzed Sandmeyer reaction gave bromide **17**, which could then undergo Suzuki–Miyaura coupling with 1,3,2-dioxaborolane **18** to afford carboxylate **19**. In a two-step process, intermediate **19** was first treated with TFA and heat to generate a pyranone intermediate that was further transformed in the presence of NH_4_OH and heat to yield pyridinone **20**. Treatment with POCl_3_ yielded chloride **21**, which could readily undergo nucleophile aromatic substitution to generate desired analogues **22**. Alternatively, chloride **21** could be subjected to an iron-catalyzed Kumada coupling with various Grignard reagents to afford analogues **23**.^42^

Select analogues **22** and **23** were screened against hM_4_ to determine potency with results highlighted in Table 2. It was evident that 2,4-dimethylpyrido[4′,3′:4,5]thieno[2,3-*d*]pyrimidine core (**6**) provided a boost in functional human hM_4_ potency. For instance, comparing the benzyl amine tail analogues **22g** (hM_4_ EC_50_ = 140 nM) and **22j** (hM_4_ EC_50_ = 170 nM) to **14j** (hM_4_ EC_50_ = 1.3 μM) and **14k** (hM_4_ EC_50_ = 1.2 μM) resulted in a 9-fold and 7-fold increase in EC_50_, respectively. This trend was also observed with small aliphatic groups. Comparing analogue **22k** (hM_4_ EC_50_ = 73 nM) to **14g** (hM_4_ EC_50_ = 290 nM) and **22f** (hM_4_ EC_50_ = 33 nM) to **14e** (hM_4_ EC_50_ = 640 nM) resulted in a 4-fold and 19-fold increase in EC_50_, respectively. Most notable was the potency difference in the pyrrolidine analogues **22e** (hM_4_ EC_50_ = 73 nM) and **14a** (hM_4_ EC_50_ > 30 μM). Taken together, these data suggest that the basicity of the nitrogen at the 7-position plays a key role in hM_4_ PAM potency and may function as a critical H-bond acceptor, while the nitrogen at the 5-position is not essential for activity.

**Table 2:**
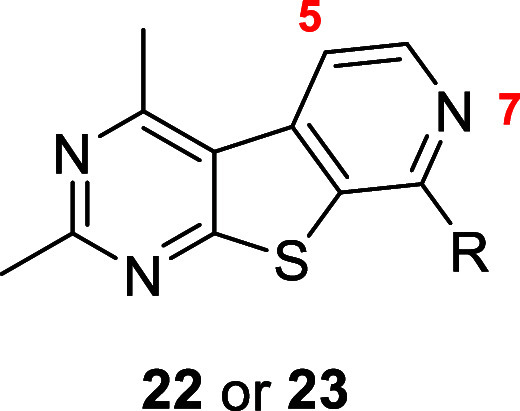

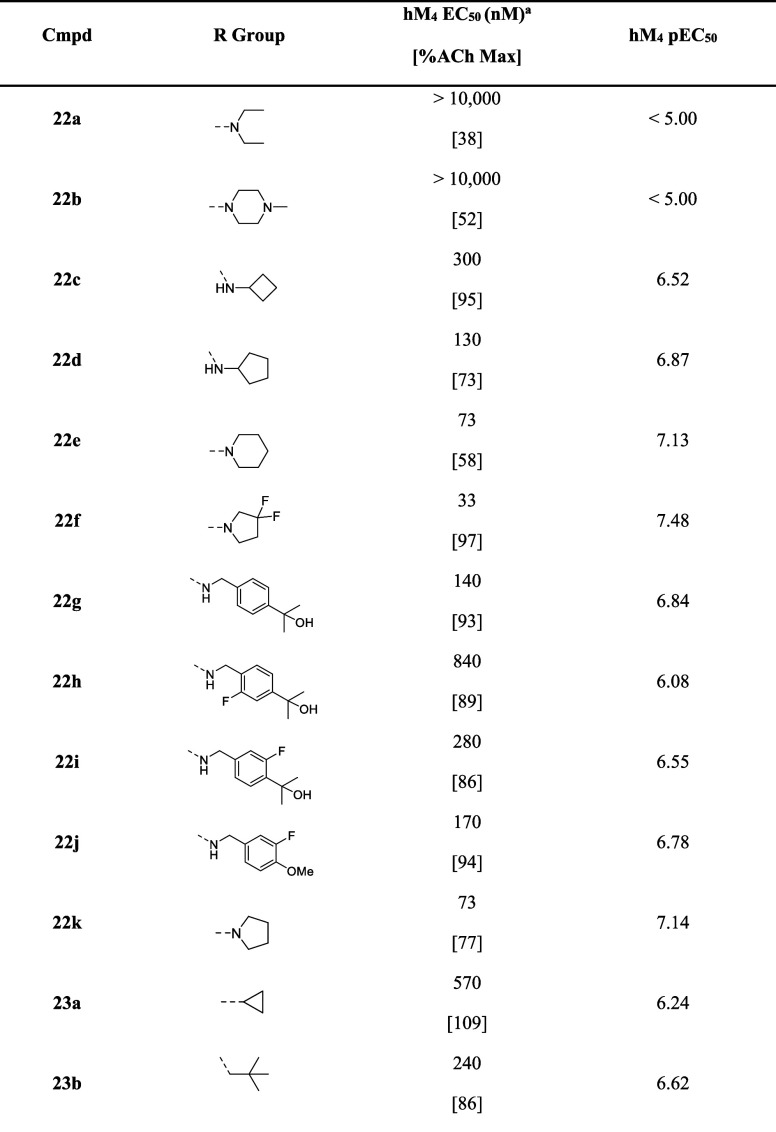
Structures and Activities for Analogues **22** and **23**

a Calcium mobilization assays with hM_4/Gqi5_-CHO cells were performed in the presence of an EC_20_ fixed concentration of acetylcholine. EC_50_ values for hM_4_ represent at least one experiment performed in triplicate.

**Scheme 3:**
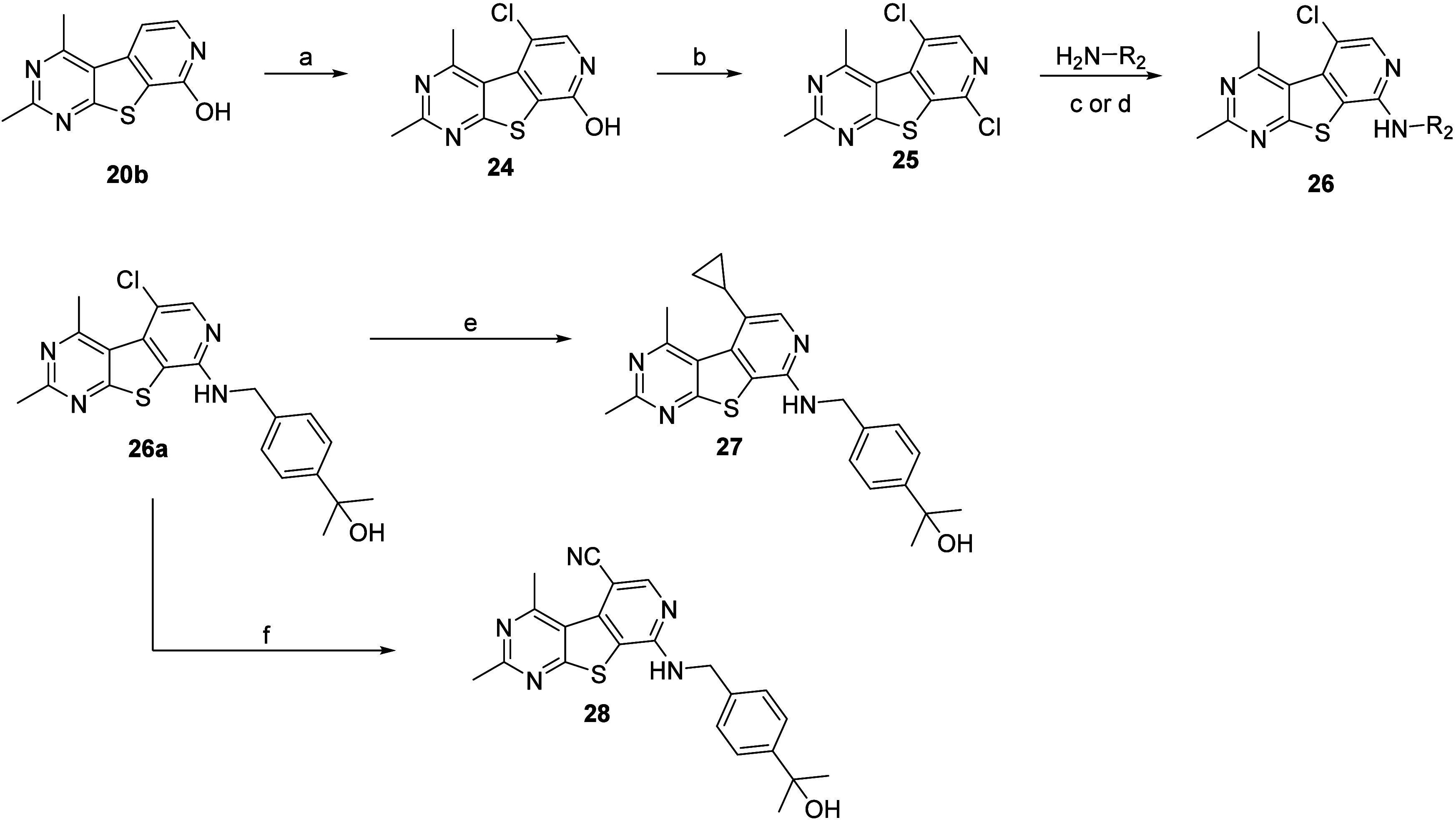
Synthesis of M_4_ PAM Analogues **26**, **27**, and **28** Reagents and conditions: (a) *N*-chlorosuccinimide (NCS), DMF, 18 h, 55%; (b) POCl_3_, microwave-irradiated at 150 °C, 1 h, 45%; (c) pyrrolidine, DIEA, NMP, microwave-irradiated at 150 °C, 30 min, 15%; (d) 2-[4-(aminomethyl)phenyl]propan-2-ol, Pd_2_(dba)_3_, Xantphos, Cs_2_CO_3_, 1,4-dioxane, microwave-irradiated at 130 °C, 1 h, 29%; (e) cyclopropylboronic acid, Cs_2_CO_3_, Pd(dppf)Cl_2_, 1,4-dioxanes/H_2_O (10:1), microwave-irradiated at 110 °C, 30 min, 11%; (f) Zn^0^, Zn(CN)_2_, NiCl_2_, dppf, 4-(dimethylamino)pyridine (DMAP), ACN, 80 °C, 2 h, 43%.

We next wanted to explore various substitutions on the pyridine ring of the tricyclic core **6** to expand upon our SAR studies, as well as elucidate the spatial constraints of the binding pocket. Being para to the amine linkage and, thus, a potential site of metabolism, we chose to explore substitutions at the 5-position of the tricyclic ring (R_2_). The preparation of analogues **27** and **28** began with chlorinating pyridinone **20b** in the presence of *N*-chlorosuccinimide to give intermediate **24** (Scheme 3). Treatment with POCl_3_ afforded dichloride **25**, which then underwent either a nucleophilic aromatic substitution or a Buchwald–Hartwig amination to generate analogues **26**. Analogue **26a** was further derivatized utilizing Suzuki–Miyaura coupling conditions to yield analogue **27**. Alternatively, analogue **26a** was subjected to palladium-catalyzed cyanation to afford analogue **28**. We decided to focus mainly on analogues containing the 2-[4-(aminomethyl)phenyl]propan-2-ol tail as this would aid in solubility, as well as the small aliphatic pyrrolidine tail for comparison.

Select analogues **22, 26, 27**, and **28** were screened against hM_4_ to determine potency with results highlighted in Table 3. It was evident that substitutions larger than methyl at the R_1_ position were tolerated; however, a decrease in the EC_50_ was observed. In fact, an increase in steric bulk [**22p** (ethyl) < **22o** (cyclopropyl) < **22n** (isobutyl)] coincided with more dramatic reductions in activity (4.0-fold, 9.3-fold, >66-fold decrease, respectively). This was also observed with the pyrrolidine analogue **22q** (>13-fold loss of potency). Similarly, substitutions at the 5-position of the ring (R_2_) also led to a loss of potency (analogues **26a**, **26b**, **27**, and **28**).

**Table 3:**
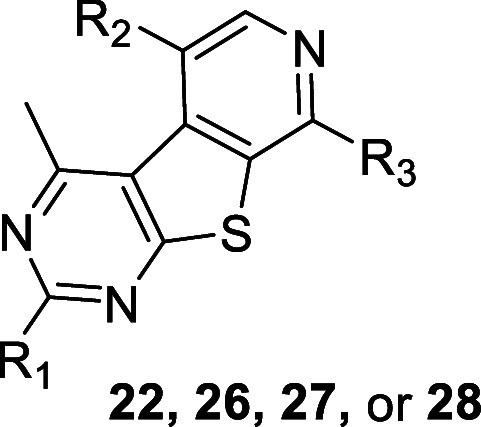

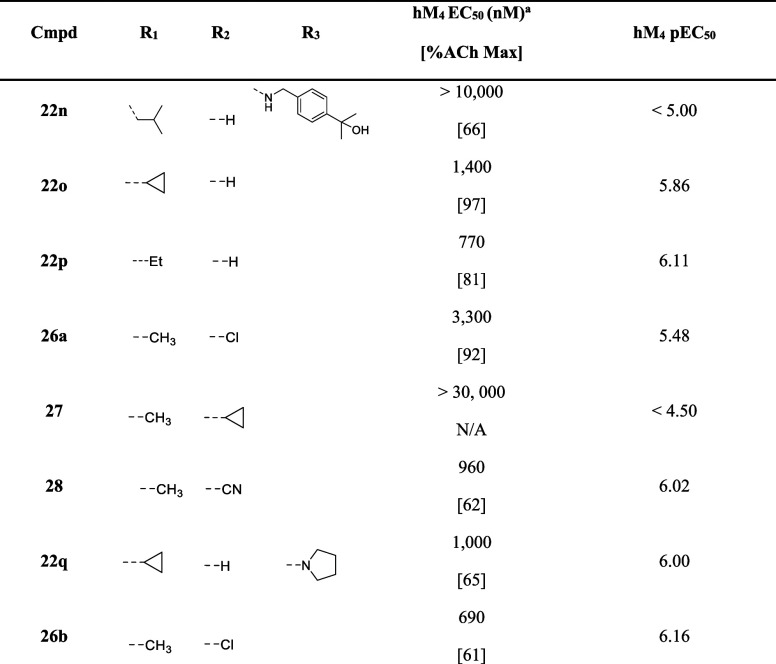
Structures and Activities for Analogues **22**, **26**, **27**, and **28**

a Calcium mobilization assays with hM_4/Gqi5_-CHO cells were performed in the presence of an EC_20_ fixed concentration of acetylcholine. EC_50_ values for hM_4_ represents at least one experiment performed in triplicate.

Of these compounds, **14h**, **14i**, **22e**, **22f**, **22g**, **22j**, and **22k** were advanced into a battery of *in vitro* drug metabolism and pharmacokinetics (DMPK) assays and our standard rat plasma-to-brain level (PBL) cassette paradigm (Table 4). These compounds were chosen to move forward based on their hM_4_ potency (EC_50_ < 190 nM), as well as their chemical diversity across subseries. In regards to physicochemical properties, these analogues all possessed molecular weights less than 400 Da and desirable topological polar surface area (TPSA) (<80 Å) with **14h**, **14i, 22e, 22f**, and **22k** having the most attractive CNS xLogP values (2.52–3.68).^43,44^ Of the analogues tested, several (**14i**, **22e**, **22f**, **22j**, and **22k**) displayed high predicted hepatic clearance in both human and rat on the basis of microsomal CL_int_ data (rat CL_hep_ ≥ 46 mL/min/kg; human CL_hep_ ≥ 15 mL/min/kg). These analogues were not progressed forward because of their high predicted hepatic clearance in rat and/or human. By contrast, analogues **14h** and **22g** display moderate human and rat predicted hepatic clearance on the basis of microsomal CL_int_ data (rat CL_hep_ of 37–44 mL/min/kg; human CL_hep_ of 12–14 mL/min/kg).

All compounds tested had low-to-moderate rat plasma protein binding (rat *f*_u,plasma_ = 1.5–13.2%). Analogue **22e** was highly bound to human plasma protein (human *f*_u,plasma_ < 1%), while all other analogues analyzed displayed moderate human plasma protein binding profiles (human *f*_u,plasma_ = 1.0–3.5%). Analogues **22e**, **22j**, and **22k** were highly bound to rat brain homogenate (*f*_u,brain_ < 1%). Conversely, analogues **14h**, **14i**, **22f**, and **22g** were moderately bound to rat brain homogenate (*f*_u,brain_ = 1.2–3.3%). All compounds tested achieved acceptable CNS penetration (*K*_p_ ≥ 1) and unbound brain to unbound plasma ratios (*K*_p,uu_ ≥ 0.76). Because of their superior DMPK profiles, **14h** and **22g** were screened against rat M_4_ (rM_4_) to ensure there was no appreciable species differences in M_4_ activity.^46^ Both analogues were potent against rM_4_ (**14h**, rM_4_ EC_50_ = 380 nM; **22g**, rM_4_ EC_50_ = 42 nM) and minor species differences were within the acceptable range (2–3.5-fold); however, **22g** was 9-fold more potent against rM_4_ than **14h**. Thus, **22g** (**VU6016235**) was selected for further evaluation in an array of *in vitro* and *in vivo* experiments.

**Table 4:** *In Vitro* DMPK and Rat PBL Data for Select Analogues **14** and **22**

property	**14h**	**14i**	**22e**	**22f**	**22g**	**22j**	**22k**
	VU6015976	VU6015368	VU6016233	VU6017369	VU6016235	VU6016234	VU6016225
MW	347.39	297.38	298.41	320.36	378.49	368.43	284.38
xLogP	3.06	2.52	3.68	3.45	4.43	4.23	3.23
TPSA	54.8	54.8	41.9	41.9	70.9	59.9	41.9
*in vitro* pharmacokinetic (PK) parameters							
CL_int_ (mL/min/kg), rat	121	1048	3444	1518	78	386	3055
CL_hep_ (mL/min/kg), rat	44	66	69	67	37	59	68
CL_int_ (mL/min/kg), human	45	177	134	148	26	50	124
CL_hep_ (mL/min/kg), human	14	19	18	18	12	15	18
rat *f*_u_,_plasma_^a^	0.132	0.048	0.019	0.043	0.024	0.014	0.038
human *f*_u_,_plasma_^a^	0.035	0.013	0.007	0.010	0.014	0.009	0.023
rat *f*_brain_^a^	0.033	0.012	0.004	0.015	0.016	0.009	0.007
brain distribution (0.25 h) (SD rat; 0.2 mg/kg IV)							
*K*_p, brain:plasma_^b^	5.07	7.27	7.77	8.33	1.14	1.69	18.6
*K*_p,uu, brain:plasma_^c^	1.27	1.82	1.64	2.91	0.76	1.01	3.43

a f_u_ = fraction unbound; equilibrium dialysis assay; brain = rat brain homogenates.

b *K*_p_ = total brain to total plasma ratio.

c *K*_p,uu_ = unbound brain (brain *f*_u_ × total brain) to unbound plasma (plasma *f*_u_ × total plasma) ratio.

**Table 5:** Further *In Vitro* Characterization of **VU6016235** (**22g**)

muscarinic selectivity^45^			
	EC_50_ (nM)	[%Ach Max]	pEC_50_
rat M_4_^a^	42	[89]	7.38
human M_1_^a^	inactive		
human M_2_^a^	6200	[46]	5.21
human M_3_^a^	inactive		
human M_5_^a^	inactive		

permeability assay			
cell line	*P*_app_ (A–B) (cm/s)	*P*_app_ (B–A) (cm/s)	efflux ratio
MDCK-MDR1	47 × 10^–6^	68 × 10^–6^	1.5

P450 inhibition IC_50_ (μM)^b^			
1A2	2D6	2C9	3A4
23	>30	17	17

a Calcium mobilization assay; EC_50_ values for rM_4_ and hM_2_ represent three experiments performed in triplicate; EC_50_ values for hM_1,3,5_ represent one to two experiments performed in triplicate.

b Assayed in pooled human liver microsomes (HLM) in the presence of NADPH with CYP-specific probe substrates.

**Figure 3:**
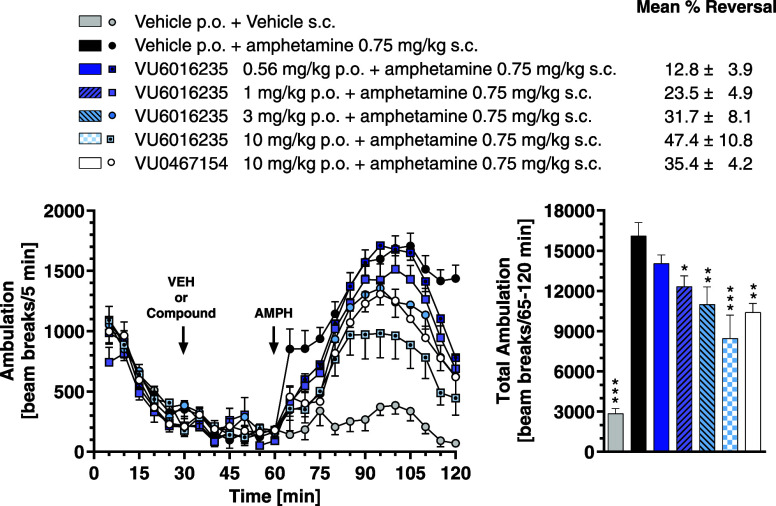
**VU6016235**, administered orally, reverses amphetamine-induced hyperlocomotion in male Sprague–Dawley rats. (Left) Time course of locomotor activity. (Right) Total locomotor activity during the 60 min period following amphetamine administration. Data are means ± SEM of 6–8 animals per group. *p < 0.05, **p < 0.01,***p < 0.001 vs Vehicle + Amphetamine. VU0467154 = positive control. Vehicle = 10% Tween80 in H_2_O.

When evaluated, **VU6016235** displayed high subtype selectivity across the mAChRs (M_1_, M_3_, and M_5_ = inactive; M_2_ = 148-fold selectivity) and was highly brain-penetrant with a P-glycoprotein (P-gp) efflux ratio of 1.5 (Table 5). Additionally, **VU6016235** displayed an excellent cytochrome P450 (CYP450) inhibition profile with IC_50_ values ≥ 17 μM across all isoforms tested (1A2, 2D6, 2C9, and 3A4). Highlighted in Table 6 are the *in vivo* rat PK parameters. **VU6016235** displayed high oral bioavailability (≥100% at a 3 mg/kg dose and 84% at a 30 mg/kg dose) and low plasma clearance (4.9 mL/min/kg) in rat. Volume of distribution was moderate (1.3 L/kg), and elimination *t*_1/2_ was 3.4 h. In a rat dose escalation pharmacokinetic (PK) study, **VU6016235** demonstrated an increase in the mean AUC_0-∞_ (3 to 30 mg/kg, po). In a preclinical pharmacodynamic (PD) model of antipsychotic-like activity, **VU6016235** demonstrated a robust dose-dependent reversal of amphetamine-induced hyperlocomotion (AHL) significant at doses of 1.0, 3.0, and 10.0 mg/kg after oral administration (MED = 1 mg/kg, Figure 3). At 90 min postdose, brain/plasma *K*_p_ and *K*_p,uu_ were determined at 1 mg/kg (*K*_p_ = 0.62; *K*_p,uu_ = 0.41), 3 mg/kg (*K*_p_ = 0.79; *K*_p,uu_ = 0.53), and 10 mg/kg (*K*_p_ = 0.82; *K*_p,uu_ = 0.55) with mean *C*_brain,u_ values of 13.9, 60.3, and 204 nM, respectively (Table 7).

**Table 6:** *In Vivo* Rat and Dog iv/po PK Profile of **VU6016235.**

iv PK					
species	dose (mg/kg)	*t*_1/2_ (hr)^b^	MRT (hr)^b^	CL_p_ (mL/min/kg)^b^	*V*_ss_ (L/kg)^b^
rat (SD)^a^	1.0	*3.4*	4.3	4.9	1.3
dog (beagle)^c^	0.5	2.4	1.6	23.8	2.3

po PK					
species	dose (mg/kg)	*T*_max_ (hr)	*C*_max_ (ng/mL)	AUC_0-∞_ (hr·ng/mL)	%*F*
rat (SD)^d^	3.0	3.0	657	8408	>100
rat (SD)^d^	30.0	5.0	4455	59164	84
dog (beagle)^e^	1.0	0.83	62.4	200	27.5

a Male Sprague–Dawley (SD) rats (*n* = 2); vehicle = 10% EtOH, 40% PEG 400, 50% Saline (1.0 mL/kg).

b *t*_1/2_ = terminal phase plasma half-life; MRT = mean residence time; Cl_p_ = plasma clearance; *V*_ss_ = volume of distribution at steady state.

c Male beagle dogs (*n* = 3); vehicle = 10% 2-hydroxypropyl-β-cyclodextrin (HP-β-CD).

d Male Sprague–Dawley rats (*n* = 2); vehicle = 10% Tween-80 in water (10 mL/kg; suspension).

e Male beagle dogs (*n* = 3); fasted; vehicle = 0.5% hydroxypropyl methylcellulose (HPMC) in water.

**Table 7:** Relationship between Total (Mean *C*_brain_) and Unbound (Mean *C*_brain,u_) Brain Concentrations of **VU6016235** and Pharmacodynamic Effects on Amphetamine (0.75 mg/kg, sc)-Induced Hyperlocomation in Rats.^a^

dose (mg/kg)	mean reversal of AHL (%)	mean *C*_plasma_^b^ (μM)	mean *C*_plasma,u_^c^ (nM)	mean *C*_brain_^b^ (μM)	mean *C*_brain,u_^d^ (nM)	brain/plasma mean *K*_p_	brain/plasma mean *K*_p,uu_
1	30.5	1.37	33.2	0.85	13.9	0.62	0.41
3	41.4	4.66	113	3.70	60.3	0.79	0.53
10	54.2	15.3	372	12.5	204	0.82	0.55

a Bioanalysis only performed for efficacious doses.

b At 1.5 h proadministration.

c Estimated unbound plasma concentration based on the rat *f*_u,p_ (0.024).

d Estimated unbound brain concentration based on the rat *f*_u,b_ (0.016).

On the basis of the encouraging *in vivo* rat PK and PD profile of **VU6016235**, the off-target and safety/toxicity profiles for this compound were further investigated. An ancillary pharmacology screen (Eurofins Panlabs) revealed no off-target activity (≤26% inhibition at 10 μM across all off-targets screened) (see the Supporting Information for the full ancillary pharmacology profile).^47^ Additionally, **VU6016235** was negative in a Mini Ames test (4 strains ± S9). Potential cardiac risks were also assessed (Charles River ChanTest Cardiac Channel Panel), and **VU6016235** was not found to strongly inhibit any cardiac ion channels tested (all ion channel inhibitions ≤14% at 10 μM (patch clamp, see the Supporting Information for additional details).^48^ With such promising data, **VU6016235** was progressed into higher species *in vivo* PK (Table 6). **VU6016235** displayed moderate oral bioavailability (27.5% at a 1 mg/kg dose) in the dog; however, high plasma clearance (∼24 mL/min/kg, nearly 78% hepatic blood flow) in the dog halted further progress of **VU6016235**.

## Conclusion

In summary, a scaffold hopping exercise utilizing a “tie-back” strategy to design M_4_ PAMs **1** and **2** proved to be a successful strategy in converting an early micromolar M_4_ PAM, **4**, into potent tricyclic M_4_ PAM analogues devoid of the classic β-amino carboxamide moiety. Two analogues within the tricycle series containing the 7,9-dimethylthieno[2,3-*d*:4,5-*d*’]dipyrimidine core core (**5**) were potent M_4_ PAMs (**14h** and **14i**; hM_4_ EC_50_ < 200 nM). Both analogues displayed favorable fraction unbound in regard to human plasma protein and rat brain homogenate binding (BHB) (1% < *f*_u_ < 5%) and low rat plasma protein binding (PPB) (*f*_u_ > 5%). Additionally, both analogues were highly CNS-penetrant (rat brain/plasma *K*_p_ > 5, *K*_p,uu_ > 1). Points of difference were observed in the predicted hepatic clearance. While **14i** displayed high human (CL_hep_ = 19 mL/min/kg) and rat (CL_hep_ = 66 mL/min/kg) predicted hepatic clearance, **14h** displayed moderate human (CL_hep_ = 14 mL/min/kg) and rat (CL_hep_ = 44 mL/min/kg) predicted hepatic clearance.

Moreover, several analogues (**22e-g** and **22j,k**) containing the 2,4-dimethylpyrido[4′,3′:4,5]thieno[2,3-*d*]pyrimidine core (**6**) were potent M_4_ PAMs (hM_4_ EC_50_ < 200 nM). Four of the five analogues profiled (**22e, 22f, 22j**, and **22k**) displayed high human (CL_hep_ ≥ 15 mL/min/kg) and rat (CL_hep_ ≥ 50 mL/min/kg) predicted hepatic clearance. Conversely, **22g** displayed moderate human (CL_hep_ = 12 mL/min/kg) and rat (CL_hep_ = 37 mL/min/kg) predicted hepatic clearance. While analogues **22e**, **22i**, and **22k** were all highly bound to rat brain homogenates (*f*_u_ < 1%), analogues **22f** and **22g** had modest fraction unbound (1% < *f*_u_ < 5%). Most analogues in this series had moderate rat and human plasma protein binding except for **22e**, which was highly bound to human plasma proteins. Furthermore, all five analogues evaluated were highly CNS-penetrant (rat brain/plasma *K*_p_ > 5, *K*_p,uu_ > 1).

Our efforts to mask the β-amino carboxamide moiety of an early M_4_ PAM compound **4** resulted in the discovery of two new tricyclic cores (**5** and **6**), which provided potent M_4_ PAM analogues that were highly brain-penetrant. This endeavor also provided analogues **14h** (**VU6015976**) and **22g** (**VU6016235**), which display 24-fold and 30-fold increase in human M_4_ activity, respectively, when compared to parent compound **4** (hM_4_ EC_50_ = 4.5 μM). Moreover, tricycles **VU6015976** and **VU6016235** exhibited moderate predicted hepatic clearance profiles in rat and human similar to parent compound **4**; this was a great improvement over previously reported tricycle **VU6007215** (Figure 2).^25^ Additionally, both **VU6015976** and **VU6016235** displayed a higher CNS distribution of unbound drug (*K*_p,uu_ = 0.76 and 1.27, respectively) compared to **VU6007215** (*K*_p,uu_ = 0.40). Overall, **VU6015976** and **VU6016235** have similarly attractive DMPK profiles; however, because of **VU6016235** exhibiting a nearly 9-fold increase in rat M_4_ activity compared to **VU6015976**, we selected **VU6016235** to carry forward for further *in vivo* PK/PD profiling. Although **VU6016235** possessed a favorable rat PK profile, displayed efficacy in a rat AHL-induced hyperlocomotion PD assay, and was determined to have minimal safety and toxicity risks, its inferior dog *in vivo* PK necessitated evaluation in alternative nonrodent species. Efforts aimed at ameliorating higher species PK are ongoing and will be reported in due course.

## Supporting Information Available

Detailed methods for the synthesis and characterization of key compounds, experimental details for all assays performed [*in vitro* pharmacology; DMPK (*in vitro* and *in vivo*); rat AHL], and additional ancillary pharmacology data (Eurofins Panlabs and Cardiac Ion Channel Panel) (PDF)

## Abbreviations

ACh: acetylcholine
AHL: amphetamine-induced hyperlocomotion
BHB: brain homogenate binding
CL_hep_: hepatic clearance
CL_int_: intrinsic clearance
CNS: central nervous system
CYP: cytochrome P450
DMPK: drug metabolism and pharmacokinetics
FDA: Food and Drug Administration
hM_4_: muscarinic acetylcholine receptor subtype 4, human
mAChR: muscarinic acetylcholine receptor
MED: minimum effective dose
PAM: positive allosteric modulator
PBL: plasma-to-brain level
PPB: plasma protein binding
rM_4_: muscarinic acetylcholine receptor subtype 4, rat
SAR: structure activity relationship
