# Supplementary material for: Discovery of VU6016235: A Highly Selective, Orally Bioavailable, and Structurally Distinct Tricyclic M4 Muscarinic Acetylcholine Receptor Positive Allosteric Modulator (PAM)
Source: ACS Chem Neurosci. 2024 Sep 24;15(20):3744–54. doi: 10.1021/acschemneuro.4c00465 (PMC11487561; doi:10.1021/acschemneuro.4c00465)
Supplement: Supplementary file 1 — cn4c00465_si_001.pdf [file cn4c00465_si_001.pdf]

Supporting Information for

Discovery of VU6016235: A Highly Selective, Orally Bioavailable, and Structurally Distinct Tricyclic M_4 Muscarinic Acetylcholine Receptor Positive Allosteric Modulator (PAM)

Julie L. Engers[a,b], Logan A. Baker[a,b], Sichen Chang[a,b], Vincent B. Luscombe[a,b], Alice L. Rodriguez[a,b], Colleen M. Niswender[a,b,d,e], Hyekyung P. Cho[a,b], Michael Bubser[a,b], Analisa Thompson Gray[a,b], Carrie K. Jones[a,b], Weimin Peng[b], Jerri M. Rook[b] Thomas M. Bridges[a,b], Olivier Boutaud[ab], P. Jeffrey Conn[a,b,d], Darren W. Engers[a,b], Craig W. Lindsley[a,b,c,d*], Kayla J. Temple[a,b*]

[a]Warren Center for Neuroscience Drug Discovery, Vanderbilt University, Nashville, TN 37232, USA
[b]Department of Pharmacology, Vanderbilt University School of Medicine, Nashville, TN 37232, USA
[c]Department of Chemistry, Vanderbilt University, Nashville, TN 37232, USA
[d]Vanderbilt Kennedy Center, Vanderbilt University School of Medicine, Nashville, TN 37232, USA
[e]Vanderbilt Brain Institute, Vanderbilt University School of Medicine, Nashville, TN 37232, USA

*Corresponding author email: kayla.temple@vanderbilt.edu
*Corresponding author email: craig.lindsley@vanderbilt.edu

Table of Contents

Experimental Synthetic Procedures and Spectroscopic Data S1
General Synthetic Methods. S2
General Instrumentation Methods. S2
General Procedure for the Preparation of Analogs 14. .... S3
General Procedure for the Preparation of Analogs 22. .... S5
Molecular Pharmacology Methods S9
Calcium Mobilization Assays. S9
DMPK Methods S10
Intravenous pharmacokinetics and plasma-brain level determination (IV PK PBL) **VU6016235** S11
Binding in plasma from rat and human. S12
Binding in brain homogenate from rat. S13
Intrinsic Clearance in Rat and Human Liver Microsomes S13
LC-MS/MS Analysis S14
Ancillary Pharmacology S15
Table S2. Lead Profiling Screen – Eurofins Panlabs for VU6016235. .... S15
Table S3. Cardiac Ion Channel Panel – Charles River Laboratories for VU6016235 .... S17
Rat Amphetamine-Induced Hyperlocomotion (AHL) Protocol S17

Experimental Synthetic Procedures and Spectroscopic Data

General Synthetic Methods.

All reactions were carried out employing standard chemical techniques. Solvents used for extraction, washing, and chromatography were HPLC grade. All reagents were purchased from commercial sources and were used without further purification.

Automated flash column chromatography was performed on a Biotage Isolera 1 or a Teledyne ISCO CombiFlash system. RP-HPLC was performed on a Gilson preparative reversed-phase HPLC system comprised of a 333 aqueous pump with solvent-selection valve, 334 organic pump, GX-271 or GX-281 liquid hander, two column switching valves, and a 155 UV detector. Absorbance was typically monitored at 215 or 220 nm. Column: Phenomenex Axia-packed Gemini C18, 5 µm. Mobile phase: CH_3CN in H_2O (0.1% TFA) or CH_3CN in H_2O (0.05% *v/v* NH_4OH) under the specified gradient, then hold 95% CH_3CN in 5% aqueous phase, 50 mL/min, 23° C. All compounds were found to be >95% pure by LCMS analysis.

Safety statement*: no unexpected or unusually high safety hazards were encountered.*

General Instrumentation Methods.

All NMR spectra were recorded on a 400 MHz AMX Bruker NMR spectrometer. ^1H and ^{13}C chemical shifts are reported in δ values in ppm downfield with the deuterated solvent as the internal standard. Data are reported as follows: chemical shift, multiplicity (s = singlet, d = doublet, t = triplet, q = quartet, b = broad, m = multiplet), integration, coupling constant (Hz).

Low resolution mass spectra (LRMS) were obtained on an Agilent 6120/6150 or Waters QDa (Performance) SQ MS with ESI source. *Method A (Agilent 6120/6150):* MS parameters were as follows: fragmentor: 70, capillary voltage: 3000 V, nebulizer pressure: 30 psig, drying gas flow: 13 L/min, drying gas temperature: 350 ºC. Samples were introduced via an Agilent 1290 UHPLC comprised of a G4220A binary pump, G4226A ALS, G1316C TCC, and G4212A DAD with ULD flow cell. UV absorption was generally observed at 215 nm and 254 nm with a 4 nm bandwidth. Column: Waters Acquity BEH C18, 1.0 x 50 mm, 1.7 um. Gradient conditions: 5% to 95% CH_3CN in H_2O (0.1% TFA) over 1.4 min, hold at 95% CH_3CN for 0.1 min, 0.5 mL/min, 55 ºC. *Method B (Agilent 6120/6150):* MS parameters were as follows: fragmentor: 100, capillary voltage: 3000 V, nebulizer pressure: 40 psig, drying gas flow: 11 L/min, drying gas temperature: 350 ºC. Samples were introduced via an Agilent 1200 HPLC comprised of a degasser,

G1312A binary pump, G1367B HP-ALS, G1316A TCC, G1315D DAD, and a Varian 380 ELSD (if applicable). UV absorption was generally observed at 215 nm and 254 nm with a 4 nm bandwidth. Column: Thermo Accucore C18, 2.1 x 30 mm, 2.6 um. Gradient conditions: 7% to 95% CH_3CN in H_2O (0.1% TFA) over 1.6 min, hold at 95% CH_3CN for 0.35 min, 1.5 mL/min, 45 ºC. *Method C (Waters QDa (Performance) SQ MS)*: MS parameters were as follows: cone voltage: 15 V, capillary voltage: 0.8 kV, probe temperature: 600° C. Samples were introduced via an Acquity I-Class PLUS UPLC comprised of a BSM, FL-SM, CH-A, and PDA. UV absorption was generally observed at 215 nm and 254 nm; 4 nm bandwidth. Column: Phenomenex EVO C18, 1.0 x 50 mm, 1.7 um. Column temperature: 55° C. Flow rate: 0.4 mL/min. Default gradient: 5% to 95% CH_3CN (0.05% TFA) in H_2O (0.05% TFA) over 1.4 min (curve 6), hold at 95% CH_3CN for 0.1 min. "Polar" (2% to 70% CH_3CN (0.05% TFA) in H_2O (0.05% TFA) over 0.8 min (curve 6), transition to 95% CH_3CN over 0.1 min (curve 6), hold at 95% CH_3CN for 0.6 min.) and "Non-Polar" (40% to 95% CH_3CN (0.05% TFA) in H_2O (0.05% TFA) over 1.4 min (curve 6), hold at 95% CH_3CN for 0.1 min.) gradients were also available. *Method D (Waters QDa (Performance) SQ MS):* MS parameters were as follows: cone voltage: 15 V, capillary voltage: 0.8 kV, probe temperature: 600° C. Samples were introduced via an Acquity I-Class PLUS UPLC comprised of a BSM, FL-SM, CH-A, and PDA. UV absorption was generally observed at 215 nm and 254 nm with a 4 nm bandwidth. Column: Phenomenex EVO C18, 1.0 x 50 mm, 1.7 um. Column temperature: 55° C. Flow rate: 0.4 mL/min. Default gradient: 5% to 95% CH_3CN in H_2O (5 mM NH_4HCO_3) over 1.4 min (curve 6), hold at 95% CH_3CN for 0.1 min. "Polar" (2% to 70% CH_3CN in H_2O (5 mM NH_4HCO_3) over 0.8 min (curve 6), transition to 95% CH_3CN over 0.1 min (curve 6), hold at 95% CH_3CN for 0.6 min.) and "Non-Polar" (40% to 95% CH_3CN in H_2O (5 mM NH_4HCO_3) over 1.4 min (curve 6), hold at 95% CH_3CN for 0.1 min.) gradients were also available.

High resolution mass spectra (HRMS) were obtained on an Agilent 6540 UHD Q-TOF with ESI source. MS parameters were as follows: fragmentor: 150, capillary voltage: 3500 V, nebulizer pressure: 60 psig, drying gas flow: 13 L/min, drying gas temperature: 275 ºC. Samples were introduced via an Agilent 1200 UHPLC comprised of a G4220A binary pump, G4226A 3 ALS, G1316C TCC, and G4212A DAD with ULD flow cell. UV absorption was observed at 215 nm and 254 nm with a 4 nm bandwidth. Column: Agilent Zorbax Extend C18, 1.8 µm, 2.1 x 50 mm. Gradient conditions: 5% to 95% CH_3CN in H_2O (0.1% formic acid) over 1 min, hold at 95% CH_3CN for 0.1 min, 0.5 mL/min, 40 ºC.

.

General Procedure for the Preparation of Analogs 14.

Synthesis of Intermediate 9 (Scheme 1):

Ammonium thiocyanate (5.86 mL, 100.0 mmol, 1.0 eq.) and acetyl chloride (7.14 mL, 100.0 mmol, 1.0 eq.) in 1,4-dioxane (100 mL) were heated at reflux for 5 minutes. Next, 3-aminocrotononitrile (8.62 mL,

100.0 mmol, 1.0 eq.) was added and the mixture was heated at reflux for 2 hours. After cooling to ambient temperature, the reaction mixture was diluted with water then extracted with $CHCl_3$/IPA (3:1, 3x). The combined extracts were washed with brine, dried over Na_2SO_4, filtered and concentrated. Purification using flash chromatography on silica gel using 0-60% EtOAc/hexanes to provided title compound (7 g, 42% yield) as an orange-brown powder. ^{1}H NMR (400 MHz, $CDCl_3$) δ 2.58 (s, 3H), 2.56 (s, 3H); LRMS: $C_7H_7N_3S$ $[M+H]^+$ calc. mass 166.0, found 166.4.

Synthesis of Intermediate 11 (Scheme 1):

To a mixture of 2-chloroacetamide (224 mg, 2.4 mmol, 1.2 eq.) and 4-mercapto-2,6-dimethylpyrimidine-5-carbonitrile (Intermediate **9**, 330 mg, 2.0 mmol, 1.0 eq) in ethanol (10 mL) was added sodium ethoxide (721 mg, 10.6 mmol, 5.3 eq.). The resulting mixture was heated at 85 °C for 3 hours. After cooling to ambient temperature, the reaction mixture was poured onto ice water. The precipitate was collected using vacuum filtration, washed with cold water and dried under vacuum to provide 175 mg of title compound as a tan powder.

The filtrate was concentrated under reduced pressure then the resulting residue was suspended in cold water. The precipitate was collected using vacuum filtration, washed with cold water and dried under vacuum to provide an additional 202 mg of title compound as a tan powder. Total yield: 377 mg, 85%. ^{1}H NMR (400 MHz, DMSO-d_6) δ 7.27 (s, 2H), 6.97 (s, 2H), 2.84 (s, 3H), 2.62 (s, 3H); LRMS: $C_9H_{10}N_4OS$ $[M+H]^+$ calc. mass 223.1, found 223.2,

Synthesis of Intermediate 12 (Scheme 1):

A suspension of 5-amino-2,4-dimethyl-thieno[2,3-*d*]pyrimidine-6-carboxamide (Intermediate **11**, 220 mg, 0.99 mmol, 1.0 eq.) in triethyl orthoformate (22.0 mL) was heated at reflux (146-150 °C). After 3 hours, the reaction mixture was concentrated to dryness under reduced pressure and azeotroped with toluene (2x) to provide the title compound as a crude material (229 mg) which was carried to the next step without further purification. ^{1}H NMR (400 MHz, DMSO-d_6) δ 8.45 (s, 1H), 3.03 (s, 3H), 2.73 (s, 3H); LRMS: $C_{10}H_8N_4OS$ $[M+H]^+$ calc. mass 233.0, found 233.4.

Synthesis of Intermediate 13 (Scheme 1):

To a solution 7,9-dimethylthieno[2,3-*d*:4,5-*d'*]dipyrimidin-4-ol (Intermediate **12**, 229 mg, 0.99 mmol, 1.0 eq.) in DCE (3.0 mL, 0.33M) at 0 °C was added triethylamine (0.412 mL, 2.95 mmol, 3.0 eq.) followed by phosphorus oxychloride (15.0 mL). The reaction mixture was stirred at 110 °C. After 3 hours, the mixture was concentrated under reduced pressure. The residue was suspended in DCM (10.0 mL) and triethylamine (0.27 mL, 1.96 mmol, 2.0 eq.) was added. The solution was filtered to remove any insoluble salts. The

filtrate was concentrated and purified using flash chromatography on silica gel using 0-50% ethyl acetate/hexanes then 50-100% EtOAc/DCM to provide the title compound as a light tan powder (200 mg, 81% yield). ^{1}H NMR (400 MHz, $CDCl_3$) δ 9.15 (s, 1H), 3.22 (s, 3H), 2.89 (s, 3H); LRMS: $C_{10}H_7ClN_4S$ $[M+H]^+$ calc. mass 251.0, found 251.3.

*Preparation of **14h (VU6015976)** (Scheme 1):*

A solution of 4-chloro-7,9-dimethylthieno[2,3-*d*:4,5-*d'*]dipyrimidine (Intermediate **13**, 25.07 mg, 0.1 mmol, 1.0 eq.), 6,6-difluoro-2-azaspiro[3.3]heptane 2,2,2-trifluoroacetate (49.43 mg, 0.2 mmol, 2.0 eq.) and DIEA (87.1 µL, 0.5 mmol, 5.0 eq.) in NMP (1.0 mL) was stirred at 50 °C for 2 h. After cooling to room temperature, the mixture was diluted with DMSO and syringe filtered to remove any insoluble salts. The crude material was purified using reverse phase HPLC to afford the title compound (27.5 mg, 79 % yield) as an off-white powder. ^{1}H NMR (400 MHz, DMSO-d_6) δ 8.67 (s, 1H), 4.51 (s, 4H), 3.07 (s, 3H), 2.96 (t, J = 12.5 Hz, 4H), 2.74 (s, 3H); ^{13}C NMR (100 MHz, DMSO-d_6) δ 169.24, 166.03, 165.22, 157.22, 155.35, 153.58, 120.02, 119.74 (t, J_{CF} = 277.5 Hz), 110.25, 60.99 (t, J_{CF} = 2.6 Hz, 2C), 44.85 (t, J_{CF} = 22, 2C), 28.21 (t, J_{CF} = 11), 25.71, 22.36; HRMS: $C_{16}H_{15}F_2N_5S$ $[M+H]^+$ calc. mass 348.1089, found 348.1089.

*Preparation of **14i (VU6015368)** (Scheme 1):*

A solution of 4-chloro-7,9-dimethylthieno[2,3-*d*:4,5-*d'*]dipyrimidine (Intermediate **13**, 6 mg, 0.02 mmol, 1.0 eq.), 3-azabicyclo[3.1.0]hexane hydrochloride (13 mg, 0.10 mmol, 5.0 eq.) and DIEA (37 µL, 0.20 mmol, 10.0 eq) in NMP (0.5 mL) was microwave irradiated for 10 minutes at 100 °C. After cooling to room temperature, the crude material was purified using reverse phase HPLC to afford the title compound (3.8 mg, 61 % yield). ^{1}H NMR (400 MHz, $CDCl_3$) δ 8.65 (s, 1H), 4.22 (d, J = 10.3 Hz, 2H), 3.89 (dt, J = 10.1, 2.2 Hz, 2H), 3.18 (s, 3H), 2.84 (s, 3H), 1.77 (dddd, J = 8.0, 4.1, 2.7, 1.5 Hz, 2H), 0.93 – 0.83 (m, 1H), 0.34 (dt, J = 5.2, 4.1 Hz, 1H); ^{13}C NMR (101 MHz, $CDCl_3$) δ 169.57, 166.49, 166.16, 157.41, 155.05, 154.92, 121.31, 112.51, 50.75 (2C), 26.36, 23.16 (2C), 16.03, 10.69. HRMS: $C_{15}H_{15}N_5S$ $[M+H]^+$ calc. mass 298.1121, found 298.1121.

General Procedure for the Preparation of Analogs 22.

*Synthesis of Intermediate **16** (Scheme 2):*

To a solution of 4-mercapto-2,6-dimethylpyrimidine-5-carbonitrile (Intermediate **9**, 10 g, 60.53 mmol, 1.0 eq.) in DMF (121 mL, 0.5 M) was added ethyl chloroacetate (8.17 mL, 72.6 mmol, 1.2 eq.) and sodium carbonate (13.1 g, 121 mmol, 2.0 eq.). The mixture was stirred at 45 °C for 3 h then heated to 90 °C for 18 h. After cooling to room temperature, the mixture was poured onto ice water. The precipitate was collected using a Buchner funnel, washed with ice cold water and dried under vacuum to provide the first batch of

the title compound as a light tan powder (13.28 g). The filtrate was extracted with EtOAc (3x). Combined extracts were dried over Na_2SO_4, filtered and concentrated. The crude material was purified using flash column chromatography on silica gel (0-50% EtOAc/DCM) to provide a second batch (567.2 mg). Total yield: 13.85 g, 91%. ^{1}H NMR (400 MHz, DMSO-d_6) δ 6.96 (s, 2H), 4.28 (q, J = 7.1 Hz, 2H) 2.86 (s, 3H), 2.63 (s, 3H), 1.29 (t, J = 7.1 Hz, 3H); LRMS: $C_{11}H_{13}N_3O_2S$ $[M+H]^+$ calc. mass 252.0, found 252.3.

*Synthesis of Intermediate **17** (Scheme 2):*

A mixture of cupric bromide (9.92 g, 44.4 mmol, 0.81 eq.) and *tert*-butyl nitrite (14.78 mL, 124.3 mmol, 2.25 eq.) in MeCN (190 mL) was stirred at rt. After 10 min, it was then slowly added to a solution ethyl 5-amino-2,4-dimethyl-thieno[2,3-*d*]pyrimidine-6-carboxylate (Intermediate **16**, 13.85 g, 55.12 mmol, 1.0 eq.) in MeCN (190 mL) via addition funnel over 1 h. After addition, the reaction mixture was poured onto water and vigorously stirred for 10 min. The precipitate was collected using a Buchner funnel, washed with cold water and dried under vacuum to provide the title compound as an off white solid (9.64 g, 56%). ^{1}H NMR (400 MHz, DMSO-d_6) δ 4.38 (q, J = 7.1 Hz, 2H) 3.05 (s, 3H), 2.71 (s, 3H), 1.34 (t, J = 7.1 Hz, 3H); LRMS: $C_{11}H_{11}BrN_2O_2S$ $[M+H]^+$ calc. mass 315.0, found 315.0/317.0.

*Synthesis of Intermediate **19** (Scheme 2):*

A mixture of ethyl 5-bromo-2,4-dimethyl-thieno[2,3-*d*]pyrimidine-6-carboxylate (Intermediate **17**, 15.7 g, 49.8 mmol, 1.0 eq.), (*E*)-1-ethoxyethene-2-boronic acid pinacol ester (21.1 mL, 99.6 mmol, 2.0 eq.), [1,1'-bis(diphenylphosphino)ferrocene]dichloropalladium (3.65 g, 4.98 mmol, 0.10 eq.), cesium carbonate (48.7 g, 149.4 mmol, 3.0 eq.) in 1,4-dioxane (301.8 mL, 0.15 M) and water (30.18 mL) was stirred at 80 °C for 18 h. The organic layer was filtered through a pad of Celite® which was washed thoroughly with EtOAc. The filtrate was diluted with water. Layers were separated. Aqueous layer wash was extracted with EtOAc (3 × 500mL). The combined extracts were dried over magnesium sulfate, filtered, and concentrated under vacuum. The crude material was purified using flash column chromatography:

Column 1: Crude material was divided equally between two 330G silica gel columns and purified using FCC (liquid loading with DCM, 330G, 5 - 25% EtOAc/DCM, 40 min). All fractions containing product were collected and concentrated to provide impure DP as an orange solid.

Column 2: Material collected from column 1 was dissolved in DCM and equally divided between two 330G silica gel columns and purified using FCC (liquid loading with DCM, 330G, 5 - 20% EtOAc/Hex, 50 min). Fractions were pooled and concentrated to provide DP as a yellow solid (8.663 g, 57%, ~90% pure by LCMS). ^{1}H NMR (400 MHz, $CDCl_3$) δ 6.71 (d, J = 13.0 Hz, 1H), 6.18 (d, J = 13.0 Hz, 1H), 4.37 (q, J = 7.1 Hz, 2H), 4.02 (q, J = 7.0 Hz, 2H), 2.85 (s, 3H), 2.78 (s, 3H), 1.40 (t, J = 7.1 Hz, 3H), 1.39 (t, J = 7.1 Hz, 3H); LRMS: $C_{15}H_{18}N_2O_3S$ $[M+H]^+$ calc. mass 307.1, found 307.2.

*Synthesis of Intermediate **20** (Scheme 2):*

A suspension of ethyl 5-[(*E*)-2-ethoxyvinyl]-2,4-dimethyl-thieno[2,3-*d*]pyrimidine-6-carboxylate (Intermediate **19**, 630 mg, 2.06 mmol, 1.0 eq.) and trifluoroacetic acid (10.8 mL) was subjected to microwave irradiation for 2 h at 120 °C. The reaction was concentrated and azeotroped with toluene to give 2,4-dimethyl-8*H*-pyrano[4',3':4,5]thieno[2,3-*d*]pyrimidin-8-one as a brown solid which was carried to the next stage without further purification. LRMS: $C_{11}H_8N_2O_2S$ $[M+H]^+$ calc. mass 233.0, found 233.4. t

To a 50 mL reinforced pressure vessel containing 2,4-dimethyl-8*H*-pyrano[4',3':4,5]thieno[2,3-*d*]pyrimidin-8-one was added ammonium hydroxide solution (6.0 mL, 28-30% NH_3 basis). The vessel was capped and heated to 100°C. After 6 h, the mixture was concentrated to dryness and azeotroped with toluene to provide the crude title compound as a pale yellow solid (475 mg, assumed quantitative yield). LRMS: $C_{11}H_9N_3OS$ $[M+H]^+$ calc. mass 232.1, found 232.2.

*Synthesis of Intermediate **21** (Scheme 2):*

A mixture of 2,4-dimethylpyrido[4,5]thieno[1,2-*b*]pyrimidin-8-ol (Intermediate **20**, 2.66 g, 11.48 mmol, 1.0 eq) and phosphorous (V) oxychloride (38 mL, 413.4 mmol, 36 eq.) was subjected to microwave irradiation at 120 °C. After 30 min, the mixture was concentrated to dryness. The crude solid was suspended in DCM and basified with neat triethylamine until pH > 8 and filtered through a filter paper to remove any insoluble salts. The filtrate was concentrated and purified using flash column chromatography on silica gel (0 - 30% EtOAc/DCM) to give a light yellow solid (852.7 mg, 30%). ^{1}H NMR (400 MHz, DMSO-d_6) δ 8.63 (d, J = 5.4 Hz, 1H), 8.33 (d, J = 5.5 Hz, 1H), 3.03 (s, 3H), 2.78 (s, 3H); LRMS: $C_{11}H_8ClN_3S$ $[M+H]^+$ calc. mass 250.0, found 250.2.

*Preparation of **22e (VU6016233)** (Scheme 2):*

A solution of 8-chloro-2,4-dimethylpyrido[4',3':4,5]thieno[2,3-*d*]pyrimidine (Intermediate **22,** 25 mg, 0.10 mmol, 1.0 eq.), DIEA (87.1 µL, 0.50 mmol, 5.0 eq.) and piperidine (49.3 µL, 0.50 mmol, 5.0 eq.) in NMP (1.0 mL) was microwave irradiated for 2 hours at 150 °C. After cooling to room temperature, the crude material was purified using reverse phase HPLC to afford the title compound (16.8 mg, 56 % yield). ^{1}H NMR (400 MHz, $CDCl_3$) δ 8.37 (d, J = 5.5 Hz, 1H), 7.57 (d, J = 5.5 Hz, 1H), 3.57 – 3.49 (m, 4H), 3.01 (s, 3H), 2.84 (s, 3H), 1.84 – 1.75 (m, 4H), 1.75 – 1.67 (m, 2H). ^{13}C NMR (101 MHz, $CDCl_3$) δ 170.51, 166.01, 163.00, 158.03, 143.98, 140.32, 122.78, 122.48, 111.88, 50.18 (2C), 26.24 (2C), 26.22, 24.85, 24.52. HRMS: $C_{16}H_{18}N_4S$ $[M+H]^+$ calc. mass 299.1325, found 299.1327.

*Preparation of **22f (VU6017369)** (Scheme 2):*

A solution of 8-chloro-2,4-dimethylpyrido[4',3':4,5]thieno[2,3-*d*]pyrimidine (Intermediate **22,** 50 mg, 0.20 mmol, 1.0 eq.), 3,3-difluoropyrrolidine (107 mg, 1.0 mmol, 5.0 eq.) and K_2CO_3 (140 mg, 1.0 mmol, 5.0 eq.) in NMP (1.0 mL) was microwave irradiated at 150 °C for 30 minutes then at 180 °C for 30 minutes. The mixture was cooled and additional 3,3-difluoropyrrolidine (107 mg, 1.0 mmol, 5.0 eq.) and K_2CO_3 (140 mg, 1.0 mmol, 5.0 eq.) were added. The mixture was then microwave irradiated at 180 °C for 2.5 hours. After cooling to ambient temperature, the mixture was diluted with warm NMP (~2 mL) and passed through a syringe filter to remove any insoluble salts then purified using reverse phase HPLC to afford the title compound (27 mg, 43 % yield). ^{1}H NMR (400 MHz, DMSO-d_6) δ 8.28 (d, J = 5.5 Hz, 1H), 7.64 (d, J = 5.6 Hz, 1H), 4.16 (t, J = 13.2 Hz, 2H), 4.07 (t, J = 7.3 Hz, 2H), 2.98 (s, 3H), 2.73 (s, 3H), 2.68 – 2.55 (m, 2H). ^{13}C NMR (101 MHz, DMSO-d_6) δ 168.99, 165.46, 163.72, 152.88, 143.93, 140.12, 128.03, 121.40, 115.48, 110.08, 54.64 (t, J_{CF} = 31.4 Hz), 46.07, 33.30 (t, J_{CF} = 23.3 Hz), 25.58, 24.29. HRMS: $C_{15}H_{14}F_2N_4S$ $[M+H]^+$ calc. mass, 321.0980, found 321.0981.

Preparation of ***22g (VU6016235)*** *(Scheme 2):*

A solution of 8-chloro-2,4-dimethylpyrido[4',3':4,5]thieno[2,3-*d*]pyrimidine (Intermediate **22,** 0.80 g, 3.2 mmol, 1.0 eq.), 2-(4-(aminomethyl)phenyl)propan-2-ol (1.06 g, 6.4 mmol, 2.0 eq.), tris(dibenzylideneacetone)dipalladium(0) (1.17 g, 1.28 mmol, 0.4 eq.), XantPhos (0.74 g, 1.28 mmol, 0.4 eq.) and cesium carbonate (3.15 g, 9.61 mmol, 3.0 eq.) in anhydrous 1,4-dioxane (32 mL) was evacuated and purged with nitrogen (3x) and stirred at 100 °C for 6 h. After cooling at room temperature, the mixture was filtered through a pad of Celite® which was rinsed thoroughly with EtOAc/DCM. Filtrate was concentrated and purified using reverse phase HPLC to afford the title compound (0.62 g, 51 % yield) as an off-white powder. ^{1}H NMR (400 MHz, DMSO-d_6) δ 8.15 (d, J = 5.6 Hz, 1H), 7.57 (t, J = 5.9 Hz, 1H), 7.44 (d, J = 5.7 Hz, 1H), 7.41 – 7.34 (m, 2H), 7.31 – 7.25 (m, 2H), 4.92 (s, 1H), 4.69 (d, J = 5.8 Hz, 2H), 2.95 (s, 3H), 2.72 (s, 3H), 1.38 (s, 6H); ^{13}C NMR (100 MHz, DMSO-d_6) δ 169.16, 164.99, 163.31, 153.01, 148.83, 144.03, 138.46, 137.93, 126.65 (2C), 124.33 (2C), 122.38, 115.20, 108.24, 70.52, 43.71, 31.99 (2C), 25.52, 23.92; HRMS: $C_{21}H_{22}N_4OS$ $[M+H]^+$ calc. mass 379.1588, found 379.1587.

Preparation of ***22j (VU6016234)*** *(Scheme 2):*

A solution of 8-chloro-2,4-dimethylpyrido[4',3':4,5]thieno[2,3-*d*]pyrimidine (Intermediate **22,** 89 mg, 0.35 mmol, 1.0 eq.), (3-fluoro-4-methoxyphenyl)methanamine (110 mg g, 0.71 mmol, 2.0 eq.), tris(dibenzylideneacetone)dipalladium(0) (49 mg, 0.053 mmol, 0.15 eq.), XantPhos (62 mg, 0.11 mmol, 0.30 eq.) and cesium carbonate (163 mg, 0.50 mmol, 1.4 eq.) in anhydrous 1,4-dioxane (1.2 mL) was evacuated and purged with nitrogen (3x) and stirred at 110 °C overnight. After cooling at room temperature, the mixture was filtered through a pad of Celite® which was rinsed thoroughly with EtOAc/DCM. Filtrate

was concentrated and purified using reverse phase HPLC to afford the title compound (49 mg, 37 % yield) as an off-white powder. ^{1}H NMR (400 MHz, DMSO-d_6) δ 8.16 (d, J = 5.6 Hz, 1H), 7.59 (t, J = 5.9 Hz, 1H), 7.46 (d, J = 5.6 Hz, 1H), 7.19 (dd, J = 12.5, 2.0 Hz, 1H), 7.13 (dd, J = 8.6, 1.9 Hz, 1H), 7.08 (t, J = 8.5 Hz, 1H), 4.64 (d, J = 5.8 Hz, 2H), 3.79 (s, 3H), 2.95 (s, 3H), 2.73 (s, 3H). ^{13}C NMR (101 MHz, DMSO-d_6) δ 169.19, 165.08, 163.44, 152.87, 145.72 (d, J_{CF} = 10.6 Hz), 144.04, 138.59, 133.58 (d, J_{CF} = 5.3 Hz), 123.30, 122.42, 115.26, 114.86, 114.68, 113.64, 108.50, 55.99, 43.11, 25.54, 23.95. HRMS: $C_{19}H_{17}FN_4OS$ $[M+H]^+$ calc. mass 369.1180, found 369.1182.

Preparation of ***22k (VU6016225)*** *(Scheme 2):*

A solution of 8-chloro-2,4-dimethylpyrido[4',3':4,5]thieno[2,3-*d*]pyrimidine (Intermediate **22,** 25 mg, 0.10 mmol, 1.0 eq.), DIEA (87 µL, 0.50 mmol, 5.0 eq.) and pyrrolidine (73 µL, 0.90 mmol, 15.0 eq.) in NMP (1.0 mL) was microwave irradiated for 1 hour at 150 °C. After cooling to room temperature, the crude material was purified using reverse phase HPLC to afford the title compound (13.5 mg, 47 % yield). ^{1}H NMR (400 MHz, $CDCl_3$) δ 8.22 (d, J = 5.5 Hz, 1H), 7.34 (d, J = 5.6 Hz, 1H), 3.94 – 3.85 (m, 4H), 3.01 (s, 3H), 2.83 (s, 3H), 2.12 – 2.00 (m, 4H); ^{13}C NMR (101 MHz, $CDCl_3$) δ 170.10, 165.55, 163.20, 154.27, 144.13, 140.23, 122.37, 116.71, 108.08, 48.75 (2C), 26.16, 25.87 (2C), 24.73. HRMS: $C_{15}H_{16}N_4S$ $[M+H]^+$ calc. mass 285.1168, found 285.1171.

Molecular Pharmacology Methods[1]

Calcium Mobilization Assays.

To measure the functional activity of positive allosteric modulator (PAM) compounds in a cellular assay, human muscarinic receptor subtype 4 (M_4) was stably co-expressed with chimeric G_{qi5} protein in the Chinese hamster ovary (CHO) cells to evoke an increase in intracellular calcium to an EC_{20} concentration of acetylcholine (ACh) agonist. The stable M_4/G_{qi5}-CHO cells were cultured in F12 medium containing 10% fetal bovine serum, 20 mM HEPES, 100 units/mL antibiotics/antimycotic, 0.5 mg/ml G418, and 0.2 mg/ml Hygromycin. All reagents used were from Life Technologies (Carlsbad, CA) unless otherwise noted.

Briefly, the day before the assay, stable M_4/G_{qi5}-CHO cells (15,000 cells/20 µL/well) were plated in black-walled, clear-bottomed, 384 well plates (Greiner Bio-One, Monroe, NC) in the culture medium without G418 and hygromycin, and then incubated overnight at 37 °C in the presence of 5% CO_2. The next day, calcium assay buffer (Hank's balanced salt solution (HBSS), 20 mM HEPES, 2.5 mM Probenecid, 4.16 mM sodium bicarbonate (Sigma-Aldrich, St. Louis, MO)) was prepared to dilute compounds, agonists, and Fluo-4-acetomethoxyester (Fluo-4-AM), fluorescent calcium indicator dye. Compounds were serially diluted 1:3 into 10-point concentration response curves in DMSO using the Bravo Liquid Handler (Agilent,

Santa Clara, CA), transferred to a 384 well daughter plates using an Echo acoustic liquid handler (Beckman Coulter, Indianapolis, Indiana), and diluted in assay buffer to a 2X final concentration. The agonist plates were prepared using acetylcholine (ACh, Sigma-Aldrich, St. Louis, MO) concentrations for the EC_{20} and EC_{MAX} responses by diluting in assay buffer to a 5X final concentration. The 2X dye solution (2.3 μM) was prepared by mixing a 2.3 mM Fluo-4-AM stock in DMSO with 10% (w/v) pluronic acid F-127 in a 1:1 ratio in assay buffer. Using a microplate washer (BioTek, Winooski, VT), cells were washed with assay buffer 3 times to remove medium. After the final wash, 20 μL of assay buffer remained in the cell plates. Immediately, 20 μL of the 2X dye solution (final 1.15 μM) was added to each well of the cell plate using a Multidrop Combi dispenser (Thermo Fisher, Waltham, MA). After cells were incubated with the dye solutions for 45 min at 37 °C in the presence of 5% CO_2, the dye solutions were removed and replaced with assay buffer using a microplate washer, leaving 20 μL of assay buffer in the cell plate. The compound, agonist, and cell plates were placed inside the Functional Drug Screening System 7000 (FDSS7000, Hamamatsu, Japan) to measure the calcium flux. After establishment of a fluorescence baseline for 2-3 seconds (2-3 images at 1 Hz; excitation, 480 ± 20 nm; emission, 540 ± 30 nm), 20 μL (2X) of test compound or vehicle was added to the cells, and the response was measured. 140 seconds later, 10 μL (5X) of an EC_{20} concentration of ACh (Sigma-Aldrich, St. Louis, MO) or vehicle was added to the cells, and the response of the cells was measured. Approximately 125 seconds later, an $EC_{80\ \mathrm{or}}$ EC_{MAX} concentration of ACh was added. Calcium fluorescence was recorded as fold over basal fluorescence and raw data were normalized to the maximal response to agonist. Calcium fluorescence was recorded as fold over basal fluorescence and raw data were normalized to the maximal response to ACh agonist. Potency (EC_{50}) and maximum response (% ACh Max) for compounds was determined using a four-parameter logistical equation using GraphPad Prism (La Jolla, CA) or the Dotmatics software platform (Woburn, MA):

$$y = bottom + \frac{top - bottom}{1 + 10^{(LogEC50 - A)Hillslope}}$$

where *A* is the molar concentration of the compound; *bottom* and *top* denote the lower and upper plateaus of the concentration-response curve; HillSlope is the Hill coefficient that describes the steepness of the curve; and EC_{50} is the molar concentration of compound required to generate a response halfway between the *top* and *bottom*.

DMPK Methods

Animal care and use: All animal study procedures were approved by the Institutional Animal Care and Use Committee and were conducted in accordance with the National Institutes of Health regulations of animal care covered in Principles of Laboratory Animal Care (National Institutes of Health).

In-life phase

Intravenous pharmacokinetics and plasma-brain level determination (IV PK PBL) **VU6016235**

Compounds were formulated as a solution in ethanol, PEG400, and saline (1:4:5 v/v, respectively) at a concentration of 1 mg/mL and administered as a single 0.2 mg/kg IV dose (1 mL/kg) to male, Sprague Dawley rats (n = 1; 342 gram body weights) via injection into a surgically-implanted jugular vein catheter. For dog PK, **VU6016235** was formulated in 10% (2-Hydroxypropyl)-beta-cyclodextrin (HPbCD) at a concentration of 0.5 mg/ml and administered as a single dose of 0.5 mg/kg to male beagles (1 ml/kg, n=3). Blood samples were collected serially from a surgically implanted carotid artery catheter in each animal over multiple post-administration time points (0.033, 0.117, 0.25, 0.5, 1, 2, 4, 7, and 24 hours) into chilled, K2EDTA anticoagulant-fortified tubes and immediately placed on wet ice. The blood samples were then centrifuged (1700 rcf, 5 minutes, 4 °C) in order to obtain plasma samples, which were stored at -80 °C until analysis by LC-MS/MS.

For determination of the brain over plasma ratio (K_p), compounds were formulated in 8% ethanol, 32% PEG400 and 60% DMSO (v/v/v) and administered as a single 0.2 mg/kg IV dose (1 mL/kg) to male, Sprague Dawley rats (n = 1; 316 gram body weights) via injection into a surgically-implanted jugular vein catheter. At 15 min post dosing, blood sample was collected serially (i.e., terminally) into chilled, K2EDTA anticoagulant-fortified tube and immediately placed on wet ice. The blood sample was then centrifuged (1700 rcf, 5 minutes, 4 °C) to obtain plasma sample. At the same post-administration time point, whole brain sample was obtained by rapid dissection, rinsed with PBS, and immediately frozen in individual tissue collection box (dry ice). All brain and plasma samples were stored at -80 °C until analysis by LC-MS/MS.

For PO PK studies, **VU6016235** was formulated in 10% Tween 80 in water and administered orally as a single dose of 10 mg/kg to male Sprague Dawley rats (10 ml/kg, n=2) that were fasted overnight. For dog PK, **VU6016235** was formulated in 0.5% hydroxypropylmethylcellulose (HPMC) in water and administered orally as a single dose of 1 mg/kg to male Beagles (5 ml/kg, n = 3) that were fasted overnight. Blood samples were collected serially from a surgically implanted carotid artery catheter in each animal over multiple post-administration time points (0.25, 0.5, 1, 2, 4, 7, and 24 hours) into chilled, K2EDTA anticoagulant-fortified tubes and immediately placed on wet ice. The blood samples were then centrifuged (1700 rcf, 5 minutes, 4 °C) in order to obtain plasma samples, which were stored at -80 °C until analysis by LC-MS/MS.

Samples preparation for bioanalysis

Plasma samples from the in-life phase of the study were thawed at ambient temperature (benchtop), and then aliquots (20 μL per sample) were transferred to a 96-shallow-well (V-bottom) plate. Matrix-

matched quality control (QC) samples and a standard curve of **VU6067104** (1 mg/mL DMSO stock solution) were prepared in blank rat plasma (K2EDTA-treated) or blank brain homogenate via serial dilution and transferred (20 µL each) to the plate along with multiple blank plasma and brain homogenate samples. Acetonitrile (120 µL) containing IS (10 nM carbamazepine) was added to each well of the plate to precipitate protein. The plate was then centrifuged (4000 rcf, 5 minutes, ambient temperature), and resulting supernatants (60 µL each) were transferred to a new 96-shallow-well (V-bottom) plate containing an equal volume (60 µL per well) of water (Milli-Q purified). The plate was then sealed in preparation for LC-MS/MS analysis.

Preparation of brain samples was identical to that of plasma samples except for the following modifications. While thawing, brains were weighed (inside their collection boxes using a universal empty collection box tare weight) and then subjected to mechanical homogenization (Mini-BeadBeater™, BioSpec Products, Inc., Bartlesville, OK) in the presence of zirconia/silica beads (1.0 mm) and extraction buffer (isopropanol:water, 7:3, v/v; 3 mL per sample, corrected for post-quantitation). Homogenized brain samples were then centrifuged (4000 rcf, 5 minutes, ambient temperature), and 5 µL of the supernatant was diluted in 15 µL of blank plasma for quantification of the analyte. The plasma standard curve and QCs were used for compounds quantitation in brain.

<u>Binding in plasma from rat and human.</u>

Determination of compounds' fraction unbound (f_u) in plasma from rat and human was conducted *in vitro* via equilibrium dialysis using HTDialysis membrane plates. Dialysis membranes (four paired strips per HTD assay) were hydrated as described by the manufacturer and inserted into the HTD plate, which was assembled and prepared for sample addition by the dispensing of blank buffer (DPBS, 100 µL/well) into the 'top half' of each membrane-split well. Each compound was diluted into plasma from each species (5 µM final concentration), which was aliquoted in triplicate to the 'bottom half' of the prepared HTD plate wells. The HTD plate was sealed and incubated for 6 hours at 37 °C. Following incubation, each well (both top and bottom halves) were transferred (20 µL) to the corresponding wells of a 96-shallow-well (V-bottom) plate. The daughter plates were then matrix-matched (buffer side wells received equal volume of plasma, and plasma side wells received equal volume of buffer), and extraction solution (120 µL; acetonitrile containing 50 nM carbamazepine as IS) was added to all wells of both daughter plates to precipitate protein and extract test article. The plates were then sealed and centrifuged (3500 rcf) for 10 minutes at ambient temperature. Supernatant (60 µL) from each well of the daughter plates was then transferred to the corresponding wells of new daughter plates (96-shallow-well, V bottom) containing water (Milli-Q, 60 µL/well), and the plates were sealed in preparation for LC-MS/MS analysis (see below).

f_u was calculated as (analyte to IS MS peak area ratio from Trans-buffer side) / (analyte to IS MS peak area ratio from Cis-plasma side). Mean values for each species were calculated from 3 replicates.

Binding in brain homogenate from rat.

Determination of fraction unbound (f_u) in brain homogenate from rat was conducted using the same methodology and procedure than described for plasma protein binding assay with the following modifications: 1) a final compound concentration of 1 μM was used, 2) naïve rat brains were homogenized in DPBS (1:3 composition of brain: DPBS, w/w) using a Mini-Bead Beater™ machine in order to obtain brain homogenate.

The diluted fraction unbound (f_{u2}) in brain was calculated as (analyte to IS MS peak area ratio from Trans-buffer side) / (analyte to IS MS peak area ratio from Cis-brain homogenate side). Undiluted fraction unbound for the brain was calculated using the following equation:

$$f_u = \frac{1/4}{\left\{\left(\frac{1}{f_{u2}}\right) - 1\right\} + 1/4}$$

Mean values for each species were calculated from 3 replicates.

Intrinsic Clearance in Rat and Human Liver Microsomes

The *in vitro* intrinsic clearance (CL_{int}) was investigated in commercially obtained hepatic microsomes from rat and human donors using the substrate depletion (i.e., loss-of-parent vs. time, or $t_{1/2}$ method) approach with analyte detection via liquid chromatography-tandem mass spectrometry (LC-MS/MS). For each species, mean %parent remaining values at each time point were calculated from replicates raw data (analyte:IS peak area ratios) and used to determine *in vitro* $t_{1/2}$ and CL_{int}.

Experiments were carried out using a robot-assisted (TECAN model Evo 200). Compound was incubated (1 μM final concentration) in buffer (100 mM potassium phosphate pH 7.4 with 3 mM $MgCl_2$) containing hepatic microsomes (0.5 mg/mL final concentration) from multiple species, discretely, at 37 °C under constant orbital shaking. After 5 minutes (pre-incubation), reactions were initiated by addition of nicotinamide adenine dinucleotide phosphate (NADPH, 1 mM final concentration). At selected time intervals (0, 3, 7, 15, 25, and 45 minutes) post-addition of NADPH, aliquots (50 μL) were taken and placed into a 96-shallow-well plate containing ice cold acetonitrile (150 μL) with carbamazepine (IS, 50 nM). The plates were then centrifuged (3000 rcf at 4 °C) for 10 minutes. The supernatants were transferred to a new 96-shallow-well daughter plate and diluted (1:1 v/v) with water (Milli-Q filtered). The plates were then sealed in preparation for LC-MS/MS analysis (see below).

Raw LC-MS/MS peak area data generated from the assay samples were used to construct natural log-transformed %parent remaining vs. time plots (using t = 0 minute post-NADPH addition sample data as starting point set to 100%). *In vitro* compound half-life ($t_{1/2}$) values were obtained using the following equation:

$$t_{1/2} = \frac{Ln(2)}{k}$$

Where k is the slope from linear regression analysis of the natural log-transformed data (using means from all replicates at each time point). Resulting $t_{1/2}$ values were then used to calculate hepatic CL_{int} values according to the following equation and with the use of species-specific scale-up factors for liver weight (grams) per total body weight (kg):

$$CL_{int} = \frac{0.693}{in\ vitro\ t_{1/2}}\ x\ \frac{1\ mL\ incubation}{0.5\ mg\ microsomes}\ x\ \frac{45\ mg\ microsomes}{1\ gram\ liver}\ x\ \frac{^{a}\ gram\ liver}{kg\ body\ wt}$$

[a]Scale-up factors used are 45 (rat) and 20 (human).[2]

Predicted hepatic clearance (CL_{hep}) was calculated using the following equation:

$$CL_{hep} = \frac{Q_h * CL_{int}}{Q_h + CL_{int}}$$

Q_h represents hepatic blood flow (mL/min/kg): 21 for human, 70 for rat, and 90 for mouse.

LC-MS/MS Analysis

Prepared samples were injected (10 µL each) onto an AB Sciex Triple Quad 4500 mass spectrometer system with an Agilent 1260 Infinity II pump and autosampler. Mass spectrometer conditions are described in **Table S1.** Quantitation of compounds was performed via AB Sciex Multiquant software using the raw analyte:IS peak area ratios. The typical detection range was 0.5 ng/mL to ≥ 5,000 ng/mL utilizing a quadratic equation regression with 1/x2 weighting.

Correction for dilution of all brain samples (in extraction buffer and subsequently in blank plasma, as previously described) was performed post-quantitation. The corrections for dilution in extraction buffer employed correction factors specific to each brain weight (not shown).

Table S1. **LC-MS/MS Conditions***

Injection volume	10 µL	
Mobile phase A	0.5% Formic Acid in Water	
Mobile phase B	0.5% Formic Acid in Acetonitrile	
Flowrate	0.5 mL/min	
Gradient	Time	% Mobile Phase B
	0.0	5
	0.2	5
	0.8	95
	1.5	95
	1.7	5
	2.7	Stop

Column	Fortis C18 (50 x 3.0 mm, 3 µm)
Data collection and analysis software/version	Analyst v. 1.7.1
Ionization mode	Positive Electrospray
Curtain gas (psi)	40
GS1 (psi)	40
GS2 (psi)	40
Capillary voltage (V)	5500
Source TurboIonSpray® temp. (°C)	500

Ancillary Pharmacology

Table S2. Lead Profiling Screen – Eurofins Panlabs for **VU6016235.**

Assay Name	Species	% inh at 10 µM
Adenosine A1	hum	6
Adenosine A2A	hum	28
Adenosine A3	hum	22
Adrenergic α1A	rat	3
Adrenergic α1B	rat	7
Adrenergic α1D	hum	5
Adrenergic α2A	hum	-9
Adrenergic β1	hum	3
Adrenergic β2	hum	-5
Androgen (Testosterone)	hum	5
Bradykinin B1	hum	-1
Bradykinin B2	hum	-1
Calcium Channel L-Type, Benzothiazepine	rat	6
Calcium Channel L-Type, Dihydropyridine	rat	-1
Calcium Channel N-Type	rat	2
Cannabinoid CB1	hum	-4
Dopamine D1	hum	21
Dopamine D2S	hum	-4
Dopamine D3	hum	3
Dopamine D4.2	hum	4
Endothelin ETA	hum	-2
Endothelin ETB	hum	6
Epidermal Growth Factor (EGF)	hum	-3
Estrogen ERα	hum	-8
GABAA, Flunitrazepam, Central	rat	13
GABAA, Muscimol, Central	rat	1
GABAB1A	human	6

Assay Name	Species	% inh at 10 µM
Glucocorticoid	hum	-10
Glutamate, Kainate	rat	-8
Glutamate, NMDA, Agonism	rat	9
Glutamate, NMDA, Glycine	rat	-9
Glutamate, NMDA, Phencyclidine	rat	-2
Histamine H1	hum	-11
Histamine H2	hum	-17
Histamine H3	hum	25
Imidazoline I2, Central	rat	-1
Leukotriene, Cysteinyl CysLT1	hum	6
Melatonin MT1	hum	7
Muscarinic M1	hum	0
Muscarinic M2	hum	10
Muscarinic M3	hum	4
Neuropeptide Y Y1	hum	-1
Neuropeptide Y Y2	hum	12
Nicotinic Acetylcholine	hum	-2
Nicotinic Acetylcholine α1, Bungarotoxin	hum	-8
Opiate δ1 (OP1, DOP)	hum	-2
Opiate κ (OP2, KOP)	hum	4
Opiate μ (OP3, MOP)	hum	7
Phorbol Ester	mouse	-1
Platelet Activating Factor (PAF)	hum	7
Potassium Channel [KATP]	ham	4
Potassium Channel hERG	hum	16
Prostanoid EP4	hum	16
Purinergic P2X	rab	0
Purinergic P2Y	rat	9
Rolipram	rat	5
Serotonin (5-Hydroxytryptamine) 5-HT1A	hum	7
Serotonin (5-Hydroxytryptamine) 5-HT2B	hum	16
Serotonin (5-Hydroxytryptamine) 5-HT3	hum	-20
Sigma σ1	hum	3
Sodium Channel, Site 2	rat	3
Tachykinin NK1	hum	8
Thyroid Hormone	rat	1
Transporter, Dopamine (DAT)	hum	25
Transporter, GABA	rat	0
Transporter, Norepinephrine (NET)	hum	26
Transporter, Serotonin (5-Hydroxytryptamine) (SERT)	hum	1

Table S3. Cardiac Ion Channel Panel – Charles River Laboratories for **VU6016235**

Ion Channel	Concentration (μM)	Mean % Inhibition	Standard Deviation	Standard Error	n
hCav1.2	10	13.9	8.9	5.1	3
hCav3.2	10	6.7	2.7	1.4	4
hHCN2	10	2.6	1.9	1.1	3
hERG	10	10.9	0.9	0.4	4
hKv1.5	10	1.3	1.8	0.9	4
hKvLQT1/minK	10	4.1	5.6	2.8	4
hKv1.3	10	9	2.9	1.7	3
hNav1.5 (Tonic)	10	5.8	0.6	0.4	3
hNav1.5 (Phasic)	10	6.9	3.5	2	3

The *in vitro* effects of **VU6016235** were evaluated at room temperature using the QPatch HT® (Sophion Bioscience A/S, Denmark), an automatic parallel patch clamp system. **VU6016235** was evaluated at a concentration of 10 μM and tested in at least 3 cells ($n \geq 3$). The duration of exposure to each test article concentration was at least 5 minutes.

Rat Amphetamine-Induced Hyperlocomotion (AHL) Protocol

Male Harlan Sprague Dawley rats with a mean body weight of 240 g (range 221-264 g) were tested in SmartFrame Open Field locomotor activity test chambers to automatically record locomotor activity. All rats were habituated in locomotor activity enclosures for 30 min, followed by pretreatment by oral gavage for an additional 30 min with either vehicle or a dose of **VU6016235** or the comparator M_4 PAM **VU0467154**. Next, rats were injected subcutaneously with vehicle or a dose of 0.75 mg/kg amphetamine and then monitored for an additional 60 min. Changes in locomotor activity were recorded for a total of 120 min. Locomotor data were expressed as the number of photobeam breaks/5 min intervals across the 120-min test session or as the total ambulation, calculated as sum of photobeam beam breaks from the time of amphetamine administration (60 min) until the end of the study (120 min). Time course data were analyzed by two-way ANOVA with main effects of treatment and time; changes in total ambulation were analyzed by one-way ANOVA followed by Dunnett's *post hoc* test (GraphPad Prism 7 [GraphPad Software, San Diego, CA]). For all tests, $\alpha \leq 0.05$ was considered to represent statistical significance. Finally, percent reversal data were calculated in Microsoft Excel using the following formula: Percent Reversal = 100 - {[(total ambulation in individual animal from t = 60 to t = 120) / (mean total ambulation

from t=60 to t=120 in the VAMP group)] * 100}. Mean percent reversal ± S.E.M. was calculated for each dose group using GraphPad Prism 7.

At the end of this behavioral study, each rat was euthanized, then decapitated, and the plasma and brain tissues were collected for the evaluation of exposure levels of **VU6016235-05** or **VU0467154-9A** by pharmacokinetic analysis.

References

1) Moehle M.S.; Bender A.M.; Dickerson J.W.; Foster D.J.; Qi A.; Cho H.P.; Donsante Y.; Peng W.; Bryant Z.; Stillwell K.J.; Bridges T.M.; Chang S.; Watson K.J.; O'Neill J.C.; Engers J.L.; Peng L.; Rodriguez A.L.; Niswender C.M.; Lindsley C.W.; Hess E.J.; Conn P.J.; Rook J.M. Discovery of the First Selective M_4 Muscarinic Acetylcholine Receptor Antagonist with *in Vivo* Antiparkinsonian and Antidystonic Efficacy. *ACS Pharmacol Transl Sci.* 2021, 4, 1306-1321.
2) Lin J.H.; Chiba M.; Balani S.K.; Chen I.W.; Kwei G.Y.; Vastag K.J.; Nishime J.A. Species differences in the pharmacokinetics and metabolism of indinavir, a potent human immunodeficiency virus protease inhibitor. *Drug Metab. Dispos*. 1996, 24, 1111-1120.
